# Surgical access to the distal cervical segment of the internal carotid artery and to a high carotid bifurcation – integrative literature review and protocol proposal

**DOI:** 10.1590/1677-5449.202101931

**Published:** 2022-08-08

**Authors:** Nicolau Conte, Thais Tapajós Gonçalves, Clarina Louis, Jonas Ikikame, Adenauer Marinho de Oliveira Góes Junior

**Affiliations:** 1 Centro Universitário do Estado do Pará – CESUPA, Belém, PA, Brasil.; 2 Universidade Federal do Pará – UFPA, Belém, PA, Brasil.

**Keywords:** internal carotid artery, carotid artery diseases, mandibular osteotomy, temporomandibular joint

## Abstract

Several different maneuvers have been described for obtaining access to the distal cervical segment of the internal carotid artery or to a high carotid bifurcation. However there are different approaches to systematization of these techniques. The objective of this study is to review the techniques described and propose a practical protocol to support selection of the most appropriate technique for each case. The review is based on the results of database searches on PubMed Central, the Virtual Health Library (BVSalud), and SciELO for articles on the subject published in English or Portuguese from 1980 to 2021. Among the different maneuvers described, it appears reasonable that the first two steps should be to obtain access at the sternocleidomastoid muscle, followed by section or retraction of the digastric muscle posterior belly. If needed, temporary unilateral mandibular subluxation is an additional resource that is preferable to division of the styloid apparatus process, because of its lesser potential for morbidity. Even wider exposure can be obtained using mandibular osteotomies.

## INTRODUCTION

Surgical exposure of a high carotid bifurcation (HCB) or the distal cervical segment of the internal carotid artery (DCSICA) is challenging because of the anatomic limitations to access and the increased risk of vascular and neurological injuries. The difficulties can be even greater after traumatism, because of profuse hemorrhage and/or pulsating/expanding hematoma, in which cases rapid access is relevant to prognosis.^1^^,^^2^

Interventions involving the DCSICA or HCB can be performed using endovascular techniques and/or by open surgery. Occasionally, the procedure is not conducted on the DCSICA, but it is nevertheless necessary to expose it. The most common indications include aneurysms, tumors, atherosclerotic stenosis, and traumatisms to zone III of the neck.^3^ Regardless of the indication, maintenance of internal carotid artery (ICA) patency is always to be preferred, because it reduces the risk of neurological deficits. However, the limitations to access to the DCSICA or an HCB considerably increase the number of ICA ligations.^1^^,^^2^

Several different maneuvers for exposure of the DCSICA or an HCB have been described with the objective of reducing the difficulties and risks involved. Anterolateral approaches are most frequent, encompassing section/retraction of the sternocleidomastoid (SCM) muscle and the digastric muscle posterior belly (DMPB), division of the styloid apparatus (DSA), mandibular subluxation, and osteotomies.^3^^,^^4^ In turn, posterolateral approaches, such as mastoidectomy, are less common and are normally reserved for when there is involvement of the petrous portion of the ICA.^5^

Although some clinical^3^^,^^6^ and experimental studies^6^^-^14 have proposed using a progressive sequence of these techniques according to the need for additional exposure of the DCSICA or an HCB, there are disagreements with regard to the sequence of the procedures. Moreover, these studies do not highlight relevant practical issues, such as decision-making time, the hospital resources needed, and surgeon expertise.

Unfortunately, none of the maneuvers described for surgical exposure of the DCSICA or HCB is free from the potential for complications and a lack of knowledge on the part of the professionals who execute them increase the morbidity of these procedures. The objective of this study is to review the techniques that have been described to date, collecting them in a single article and proposing a practical protocol to support the choice of the most appropriate technique for each case.

## METHODS

An integrative literature review was conducted of published studies indexed on the PubMed Central, SciELO, and Virtual Health Library (BVSalud) databases. The following keywords were used: internal carotid artery, distal, cervical, extracranial, carotid bifurcation, high, open surgery, open approach, exposure, access, subluxation, luxation, osteotomy, mandibular, styloid, and digastric. These terms were adapted to suit the language of the databases being queried and combined using Boolean operators to refine the searches.

Studies were selected according to the following inclusion criteria: 1) clinical and experimental studies that describe techniques used to expose the carotid; 2) injuries involving the DCSICA or HCB; 3) published from January 1980 to September 2021; and 4) written in English or Portuguese. Publications were excluded that 1) employed endovascular or hybrid approaches; 2) required exposure of the petrous portion of the ICA; 3) described exclusively posterolateral approaches; or 4) for which the full text was unavailable.

Articles not selected for data tabulation were nevertheless used as references for the discussion. Additionally, Brazilian and international specialists were contacted to request images from real cases, previously presented/published material, and authorization to use them for illustration in this study.

## RESULTS

**Figure 1:**
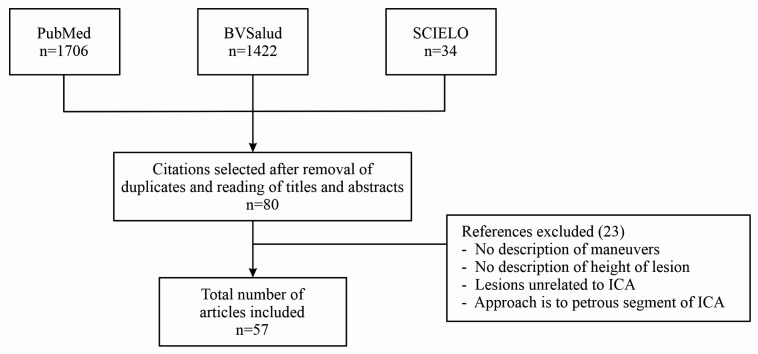
Flow diagram illustrating selection of articles. ICA: internal carotid artery.

The initial searches using the keywords chosen returned 3,162 articles (Figure 1). All duplicate articles were removed and then the inclusion and exclusion criteria were applied in all of the analytical stages. The first stage comprised reading of titles and abstracts, which resulted in selection of 80 articles and the second stage comprised reading of the full texts of articles for which they were available, resulting in inclusion of 57 articles in the review. Nine of these were experimental,^7^^-^14 47 were clinical studies (case reports and series),^3^^,^^15^^-^57 and one^6^ was a clinical and experimental study. The total sample included 101 specimens (cadaveric carotid arteries dissected) and 480 patients (see Tables S1 and S2, available online as a supplementary file), and the most frequent indications for exposure of DCSICA or HCB were stenosis due to atheromatosis and aneurysms (see Table S2, available online as a supplementary file).

## DISCUSSION

### Surgical anatomy and anatomic variations

The cervical segment of the ICA starts at the level of the bifurcation of the common carotid artery (CCA) and ends at the level of the carotid foramen. The bifurcation normally occurs at the level of C3/C4, posterior to the superior margin of the thyroid cartilage.^58^ However, significant anatomic variations have been described, including bifurcation at the levels of T4 and Cl.^59^ By convention, the carotid bifurcation is defined as “high” if it occurs cranial of C3/C4, above the hyoid bone/thyroid cartilage^58^^,^^60^ or above the Blaisdell line (a line traced between the tip of the mastoid process and the angle of the mandible)^27^ and “low” if it occurs below these anatomic references.^58^^,^^60^

After the CCA bifurcation, the ICA follows an ascending path within the carotid sheath, posteromedial of the internal jugular vein and the vagus nerve, to the carotid canal in the temporal bone.^61^^,^^62^ There are variations in the course the vessel takes along this path, which can be classified into four categories: straight (type 1), curved (type 2), kinking (type 3), or loop (type 4).^60^

For the purposes of surgical planning, it is relevant to divide the cervical portion of the ICA into proximal and distal segments. Several different anatomic landmarks have been described as references for this division, including the level at which the hypoglossal nerve crosses the ICA, the Blaisdell line, and the DMPB.^3^^,^^5^^,^^9^ The proximal segment extends from the CCA bifurcation to one of these landmarks and the distal segment comprises the portion of the ICA from these structures to the base of the skull.^61^^,^^62^ Anatomic structures related to approaches to the ICA are listed in Table 1.

**Table 1:** Principal structures found in the topography of access to the internal carotid artery.^61^^,^^62^

**Structure**	**Anatomic landmarks**	**Repercussions of injury**
Nerve V_3_ (trigeminal/ mandibular ramus)	Emerges from the skull through the foramen ovale. Its downward path accompanies the inferior alveolar artery, medial to the mandibular ramus. It continues deep to the lateral pterygoid muscle and then between the sphenomandibular ligament and the ramus of the mandible to the mandibular foramen.	Paresthesia of the teeth of the mandible, the region of the mentum, and the lower lip.
Nerve VII (facial)	Emerges from the base of the skull at the stylomastoid foramen. It runs at the same depth or slightly deeper than the superior margin of the digastric muscle posterior belly. It is accompanied by the stylomastoid artery. It has a relationship with the tympanomastoid fissure, which is from 7 to 15 mm lateral to the exit of the VII cranial nerve at the stylomastoid foramen.	Facial paralysis.
Nerve IX (glossopharyngeal)	It emerges from the skull at the jugular foramen. It runs medial of the styloid process and lateral of the stylopharyngeus muscle and the internal carotid artery. It continues caudally posterior to the stylopharyngeus muscle. Anteriorly, it is located between the pharyngeal constrictor muscles (superior and middle).	Loss of sensitivity in the posterior third of the tongue; reduced parotid gland secretion; Vernet syndrome; Collet Sicard syndrome.
Nerve X (vagus)	Emerges from the skull at the jugular foramen. The superior portion runs posterior and medial to the internal carotid artery. The inferior portion runs between the common carotid and internal jugular arteries. Internal branch of superior laryngeal nerve: descends to the thyrohyoid membrane, base of the tongue, epiglottic region, and mucous membrane of the larynx. External branch of superior laryngeal nerve: runs alongside the inferior pharyngeal constrictor to the cricothyroid muscle.	Paralysis of vocal cords and dysphagia (paralysis of soft palate levator muscle and pharyngeal constrictor muscles); Vernet syndrome; Collet Sicard syndrome.
Nerve XI (accessory)	Emerges from the skull at the jugular foramen. Runs between the insertion of the digastric muscle posterior belly into the mastoid process and the sternocleidomastoid muscle.	Scapular winging and shoulder droop (paralysis of the trapezius and sternocleidomastoid muscles); Vernet syndrome; Collet Sicard syndrome.
Nerve XII (hypoglossal)	Emerges from the skull at the hypoglossal foramen, running along the inferior margin of the digastric muscle posterior belly.	Unilateral paralysis and atrophy of the tongue.
Common carotid artery	Right: branch of the brachiocephalic trunk. Left: branch of the aortic arch. Runs superior and posterior to the sternoclavicular joint. Normally bifurcates at the height of the superior margin of the thyroid cartilage (C3/C4).	-
External carotid artery	Runs in an anterosuperior direction, posterior to the mandible and deep to the digastric muscle posterior belly and stylohyoid muscle.	-
Internal carotid artery	Runs cranially within the carotid sheath. Anterior of the transverse processes of the superior cervical vertebrae.	-
Internal jugular vein	Located posterior of the internal carotid artery and the glossopharyngeal, vagus, and accessory nerves until it enters the carotid sheath. Located internal of the sternocleidomastoid muscle.	-
External jugular vein	Formed in the interior of the parotid gland. Follows a descending path deep to the platysma muscle and superficial to the sternocleidomastoid.	-

### Preoperative assessment

In traumatism cases, decisions of relevance to approaches to the ICA must take hemodynamic stability and concomitant injuries into consideration.^32^ In patients who need immediate interventions, the surgeon can anticipate a need to enlarge exposure on the basis of certain criteria, such as gunshot wounds and/or injuries between the angle of the mandible and the base of the skull (cervical zone III). In contrast, in stable patients with no signs of ischemia, assessment with angiotomography is relevant and helpful for planning surgery.^31^^,^^32^

### Considerations relating to airways and patient positioning

After establishment of the airway (AW), the patient is placed in a supine position, arms along the body, and with hyperextension and contralateral rotation of the neck, facilitating more distal dissection of the ICA.^21^^,^^34^^,^^63^ However, excessive movements can cause compression of the vertebral arteries or the contralateral carotid,^64^ in addition to making SCM muscle retraction difficult because of increased muscle tension and restricting the mobility of the CCA and the carotid bifurcation.^65^

**Figure 2:**
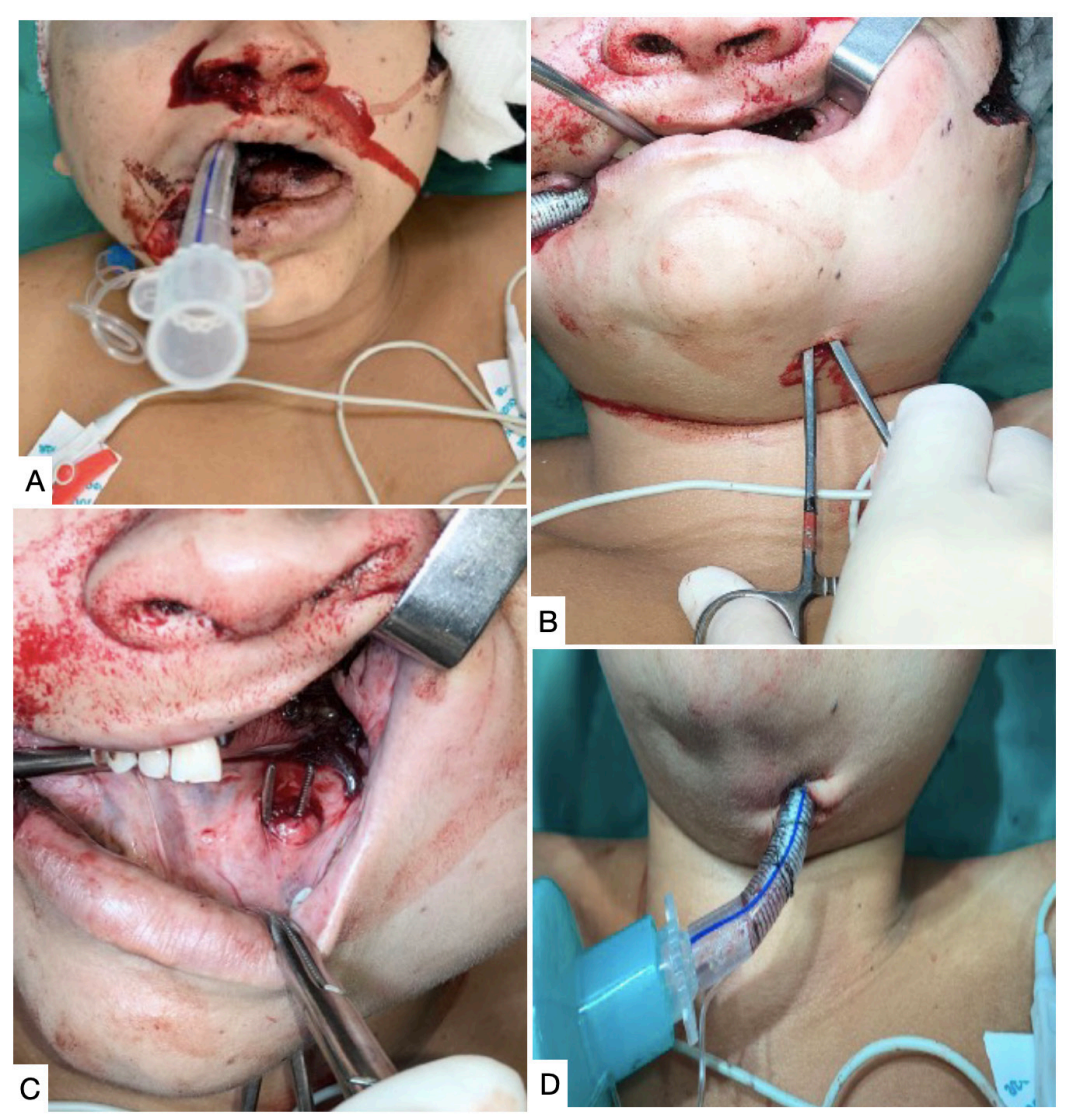
Submental conversion of oral endotracheal intubation (OEI). **(A)** Patient on OEI; **(B)** Submental access; **(C)** Dissection of the floor of the mouth; **(D)** Submental conversion of OEI.

Surgical AW and nasotracheal intubation (NTI) increase the number of possible techniques for exposure of the DCSICA or an HCB, making temporary mandibular subluxation (TMS) a viable option. If oral endotracheal intubation (OEI) is needed, TMS is only possible if the patient is edentulous or has lost several teeth. An alternative option is submental conversion of the oral endotracheal tube (OET) (Figure 2); but this maneuver increases total operating time by about 15 minutes.

It should be noted that simply maintaining the oral cavity closed after NTI does not increase the exposure afforded by the ICA access.^66^ However, combining NTI with superior traction of the maxilomandibular complex (the chin-up position), using adhesive tape or other devices, is one strategy that has been described to increase exposure of the ICA,^67^^,^^68^ although it is unnecessary if TMS is performed.

### Accesses and maneuvers used to approach the ICA or an HCB

Access to the DCSICA or an HCB is the subject of debates in the literature regarding the best sequence of maneuvers to achieve progressively distal exposure of the ICA. These maneuvers are summarized in Tables S1 and S2 (available online as a supplementary file).

First stage (conventional access): retraction of the SCM muscle (for lesions up to the level of C2)

The first step to access the ICA/carotid bifurcation consists of making skin incisions of varying dimensions and extension, depending on the subsequent stages of exposure that are planned. The majority of authors describe a vertical incision starting at the mastoid process and running parallel to the SCM muscle,^1^^,^^3^^,^^9^^-^12^,^^15^^,^^17^^,^^21^^,^^30^^,^^31^^,^^33^^,^^36^^,^^40^^,^^48^^,^^52^^,^^53^ enabling exposure of the great auricular nerve, the mastoid process, and the retromandibular fossa.^11^ Pre-auricular extensions are also described,^4^^,^^9^^,^^37^ offering the advantage of facilitating identification of the facial nerve. When mandibular osteotomies (MDO) are planned, incisions can be extended along the submandibular and submental regions and the lower lip.^12^^,^^14^^,^^30^^,^^34^^,^^37^^,^^39^^,^^43^

In addition to these structures, cutaneous branches of the cervical plexus, such as the great auricular nerve and the transverse cervical nerve, which run deep until the fascia of the SCM muscle, may also be injured during this access, causing paresthesia of the earlobe and the anterior aspect of the neck.^5^

Next, the majority of authors recommend dissection along the anterior (medial) margin of the SCM muscle for lateral retraction.^3^^,^^5^^-^17^,^^21^^,^^23^^,^^25^^-^28^,^^32^^-^38^,^^40^^,^^44^^-^50^,^^52^^-^57 The facial vein should be ligated and the hypoglossal loop can be sectioned, exposing the entire cervical portion of the CCA and a mean of 26.95 mm (varying from 15 to 45 mm)^5^ of the proximal segment of the ICA, equivalent to the level of the upper third of C2.^8^^,^^30^ However, dissections via posterior routes are also described, such as the retrojugular access^30^ and via the retromandibular fossa.^10^

It should be noted that it is possible to enlarge exposure of the ICA with some variations of the conventional technique, such as section/deinsertion of the SCM muscle at the level of the mastoid process,^7^^,^^8^^,^^18^ followed by retraction^30^^,^^31^^,^^51^ or use of a Thompson retractor,^54^ that enables vascular control 1 cm from the base of the skull.

Second stage: approach to the DMPB (for additional exposure that does not pass the superior margin of the C2)

After retraction/section/deinsertion of the SCM muscle, it is recommended that the posterior aspect of the parotid gland should be dissected in the region of the stylomastoid, where the temporoparietal fascia is sectioned after identification and careful dissection of the VII cranial nerve. This retroparotid dissection does not yield additional exposure of the ICA, but does provide a surgical field for access to the DMPB, which is an important restriction to access to the distal ICA.^5^

**Figure 3:**
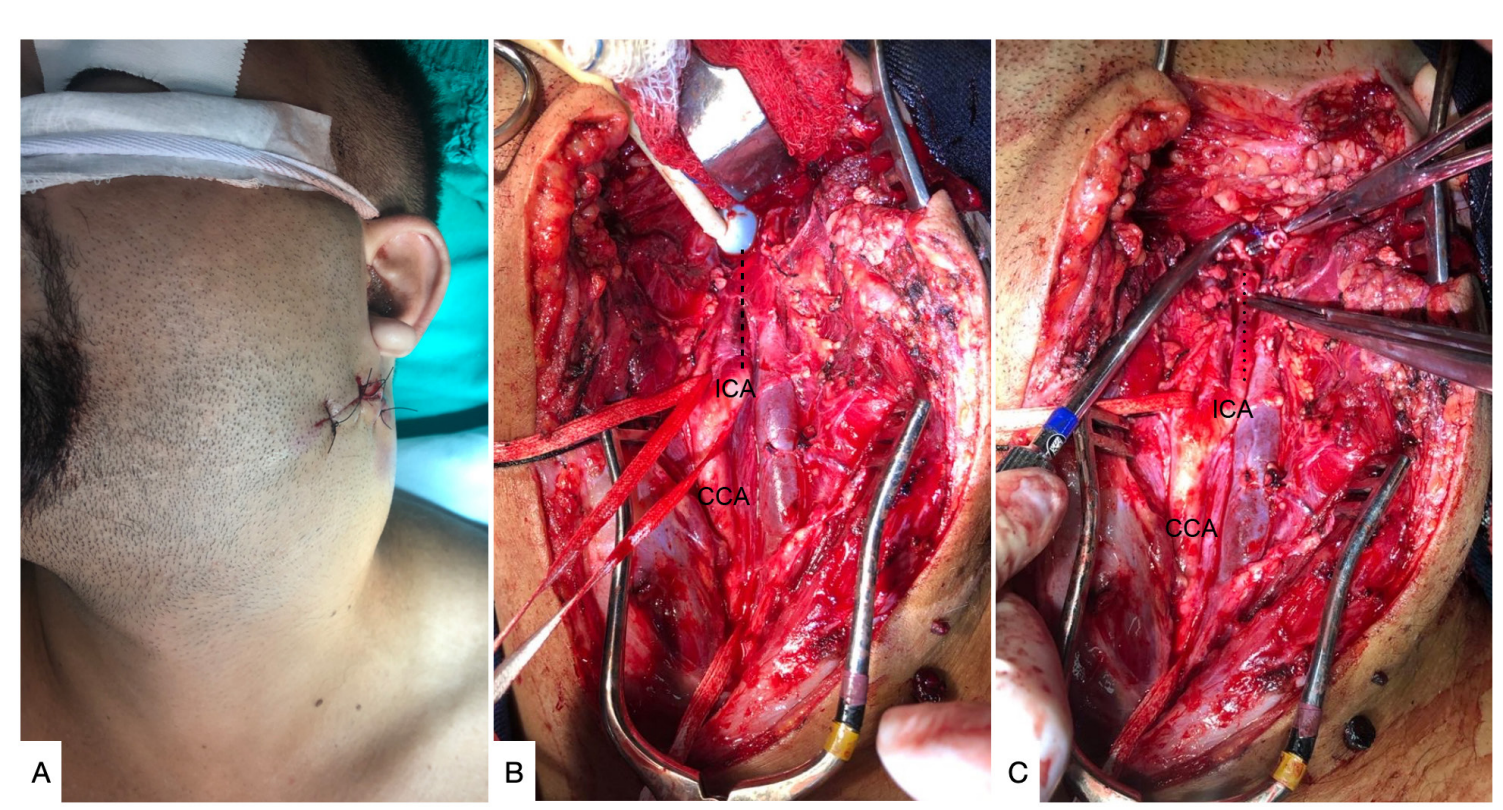
Surgical approach to a traumatic cold weapon injury in left cervical zone III. **(A)** Cervical hematoma, cutaneous plane already sutured; **(B)** Access by sternocleidomastoid muscle retraction and division of the digastric muscle posterior belly, revealing the common carotid artery (CCA) and proximal segment of the internal carotid artery (ICA). The distal segment of the ICA is not exposed (broken line). Foley catheter controlling bleeding at the base of the skull; **(C)** Exposure of the distal segment of the ICA (dotted line), distal ICA stump is clamped.

After exposure of these structure, there are differences between the recommendations contained in published studies. While some authors recommend only retracting the DMPB, the majority recommend sectioning the DMPB (Tables S1 and S2, available online as a supplementary file, and Figure 3), yielding a mean increase of 12 to 14 mm additional distal exposure of the ICA (compared to the conventional access offered by retraction of the SCM muscle).^5^^,^^6^

It should be mentioned that it is possible to avoid the DMPB by using the posterior cervical triangle proposed by Sasaki et al.^31^ This process is executed via an incision along the posterior margin of the SCM muscle, followed by longitudinal division and retraction of the muscle to both sides, making it possible to expose the region corresponding to the C1 vertebral body.

During these stages, several neurovascular structures are observed within the surgical field, including the common facial vein; the XII cranial nerve, which runs along the inferior margin of the DMPB; the XI cranial nerve, which run between the insertion of the DMPB into the mastoid process and the SCM muscle, injury to which causes scapular winging and shoulder droop; and the occipital artery, which is normally ligated to increase exposure of the ICA and facilitate dislocation of the XII cranial nerve.^5^ An important anatomic landmark for the accessory nerve (XI pair) is its lateral point, located around 3 to 15 mm below and to the side of the anterior border of the transverse process of the atlas.^7^ Additionally, medial dissection of the ICA at this level can injure the superior laryngeal nerve, causing dysphagia and dysphonia.

Third stage: TMS or DSA (for lesions up to the level of the C2)

The next anatomic limitations to access to the ICA or HCB involve the posterior mandibular region and the styloid apparatus. From this point onwards, the literature does not offer consensus on the sequence of procedures for increasing distal exposure of the ICA or HCB. It should be mentioned that the experimental studies were conducted with cadavers, exclusively evaluating the degree of exposure achieved by each maneuver and ignoring relevant factors such as the type of AW provision, the time taken for decision-making, the hospital resources available, and the expertise of the surgeon.

Some experimental studies recommend performing DSA as the next stage,^5^^,^^7^^,^^9^^,^^10^ yielding additional exposure of 10 to 15 mm;^5^^,^^9^ performing TMS only if necessary and yielding additional exposure of approximately 8 mm.^9^ However, in another cadaveric study, Mock et al.^6^ described performing TMS before DSA, yielding approximately 20 mm of additional exposure of the ICA after division of the DMPB. If necessary, DSA should be performed afterwards, yielding a further 3 mm of exposure.

Analyzing aspects inherent to TMS and DSA critically, we conclude that if TMS is possible, it should be the chosen option because of the speed and relative technical simplicity, in addition to the lower morbidity (Tables 2 and 3). Moreover, if the surgeon has the necessary expertise, TMS does not require an oral and maxillofacial surgeon.

**Table 2:** Aspects related to temporary mandibular subluxation.

**Advantages**	**Disadvantages**	**Complications**	**Time**
Low morbidity. Easy to perform. Expands the surgical field (from triangular to rectangular). Unaffected by variables such as sex, age, and length of neck.	Need for nasotracheal intubation or submental conversion of intubation. Anatomic distortion of the surgical field, with a theoretical increase in risk of hypoglossal nerve injury. Enlargement of the surgical field is limited in patients with a very steep articular eminence and a deep articular fossa.	Risk of pathological luxation of the mandible; temporomandibular dysfunction.	10-15 min

**Table 3:** Aspects inherent to division of the styloid apparatus.

**Advantages**	**Disadvantages**	**Complications**	**Time**
Permits exposure of the portion of the internal carotid artery covered by the styloid apparatus. The decision to perform this maneuver can be made intraoperatively. Can be combined with temporary mandibular subluxation to increase exposure.	Potential risk of injury to IX cranial nerve and the pharyngeal branches of the vagus nerve.	Dysphagia	-

Temporary mandibular subluxation combined with retraction of the SCM and division of the DMPB provides access to lesions of the DCSICA (above the Blaisdell line)^6^^,^^23^^,^^25^^-^28 from 3 to 4 cm below the base of the skull^23^^,^^28^ and when combined with retraction of the styloid muscles, reaches a point 1 cm proximal of the base of the skull.^15^ If it is still necessary to achieve additional ICA exposure after the TMS, we suggest performing DSA, thereby avoiding exposing the patient to the surgical risks inherent to this technique when the DCSICA and HCB can be adequately exposed by TMS.

### TMS

This is the technique described by the majority of studies for elective procedures in patients with HCB or cases in which it is necessary to access the distal ICA for treatment of atherosclerotic disease, tumors, fibromuscular dysplasia, pseudoaneurysms, aneurysms, or arteriovenous fistulas, although there are also reports of its use in acute trauma cases (Table S2, available online as a supplementary file). Temporary mandibular subluxation is a safe, rapid (10-15 minutes), and relatively easy procedure,^3^^,^^9^ in which anterior subluxation of the condyle (10 to 15 mm) at the articular eminence results in anterior displacement of the mandibular ramus by 20 to 30 mm, increasing distal ICA exposure by transforming a triangular operating field into a rectangle.^3^ With the exception of the study by Mock et al.,^6^ in which TMS was performed bilaterally, all other studies (clinical and cadaveric) employed the unilateral technique, without restricting carotid exposure.

To enable TMS, it is necessary to employ NTI or OEI with submental conversion. Ideally, TMS should be performed soon after the AW is established (when the need for TMS can be predicted), although it can be performed after the initial surgical accesses described above. On this point, it is worth mentioning that TMS causes a slight anatomical distortion, displacing the DMPB and hypoglossal nerve forwards and upwards, in addition to provoking medial rotation of the carotid bifurcation. Dissection must therefore be conducted more cautiously to avoid injury to the hypoglossal nerve.^9^

**Figure 4:**
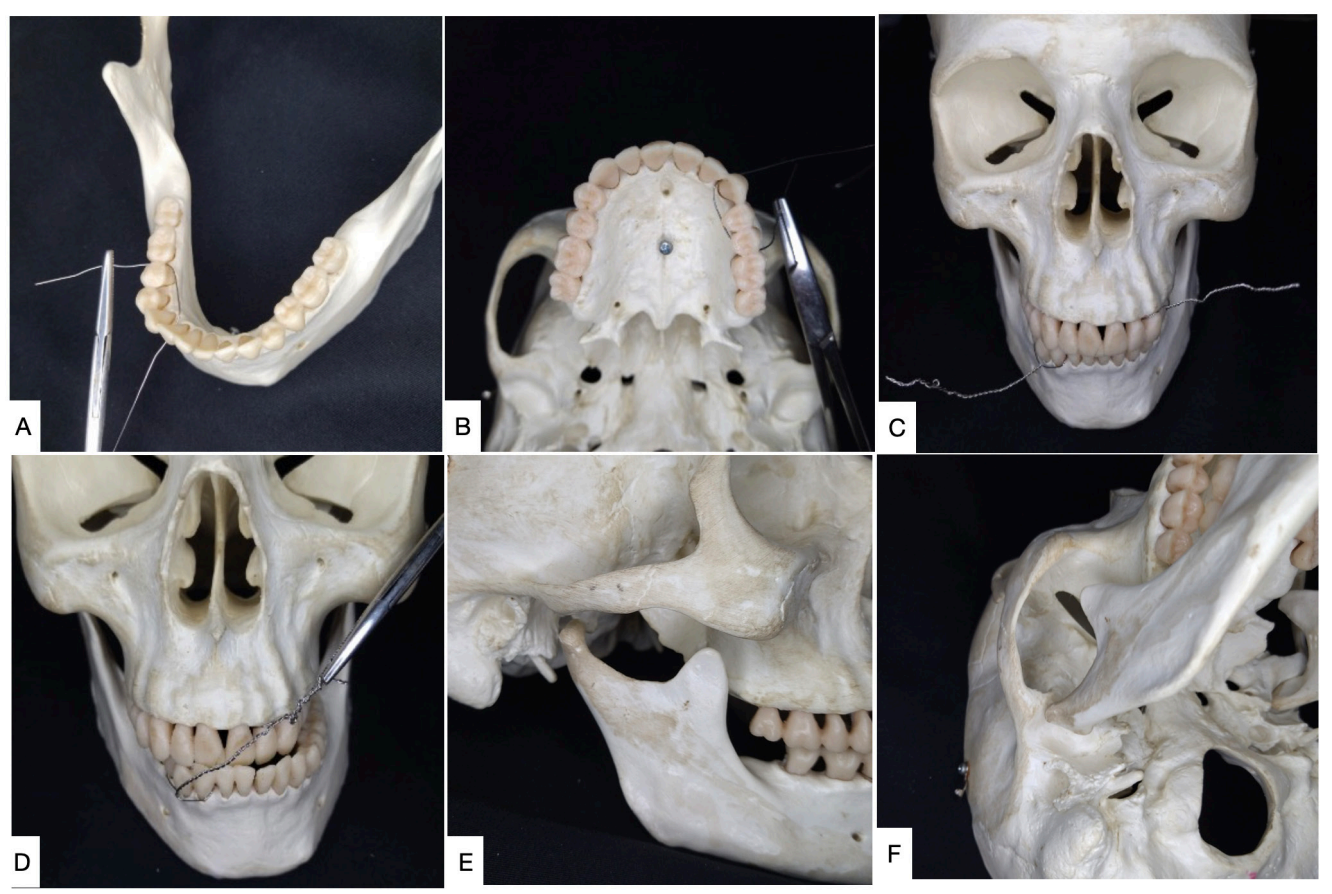
Technique for mandibular subluxation with steel wires. **(A)** Positioning steel wire in mandibular interdental spaces; **(B)** Positioning steel wire in maxillary interdental spaces; **(C)** Twisting steel wires individually; **(D)** Twisting crossed steel wires to achieve mandibular subluxation; **(E)** Luxated mandibular condyle in lateral view; **(F)** Luxated mandibular condyle in axial view.

**Figure 5:**
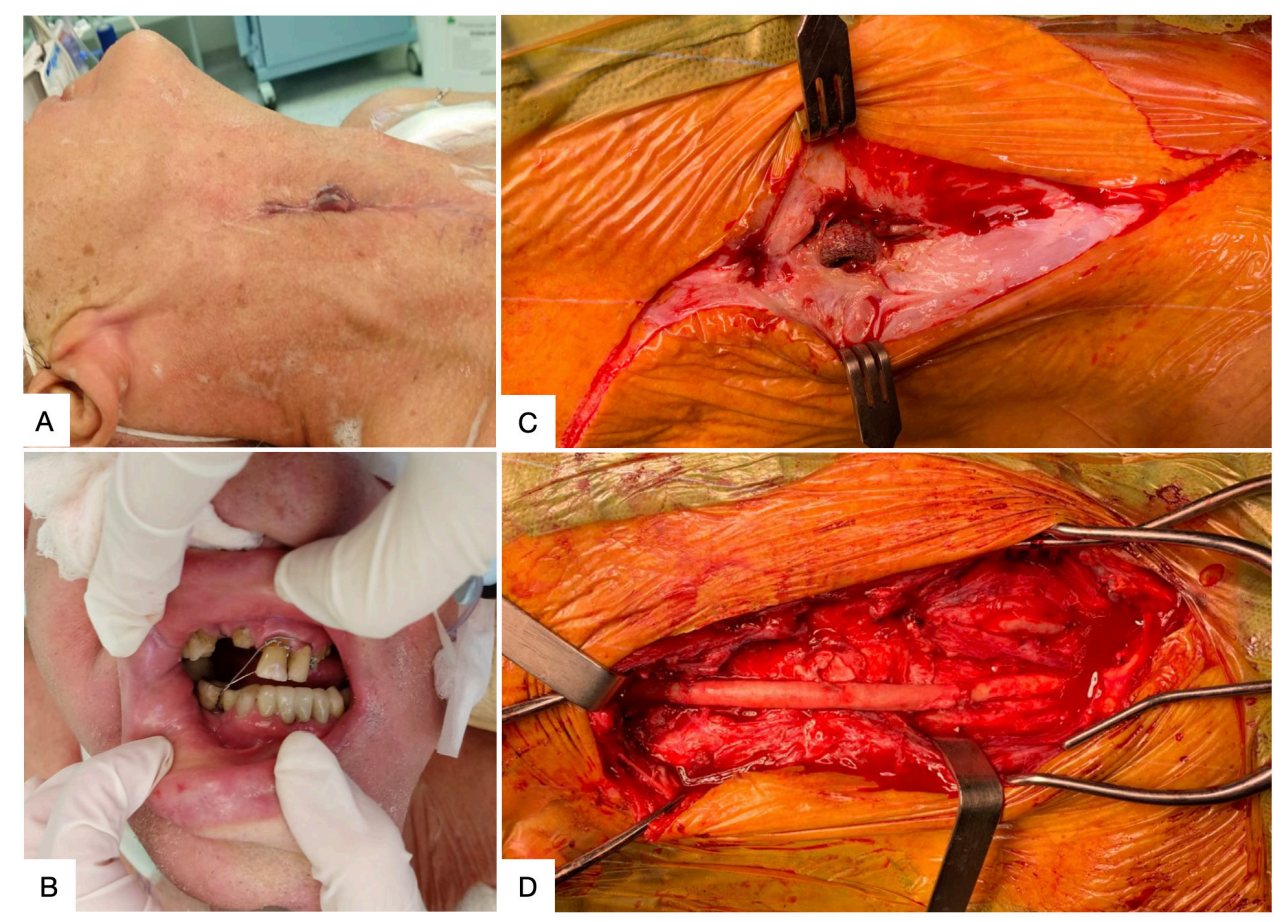
Application of TMS to obtain access to a contaminated stent in the internal carotid artery. **(A)** Infection fistularizing at the level of the surgical scar; **(B)** Mandibular subluxation with interdental wires; **(C)** Access via sternocleidomastoid muscle retraction and division of the digastric muscle posterior belly; **(D)** Internal carotid artery reconstructed with an autologous graft (femoral artery).

**Figure 6:**
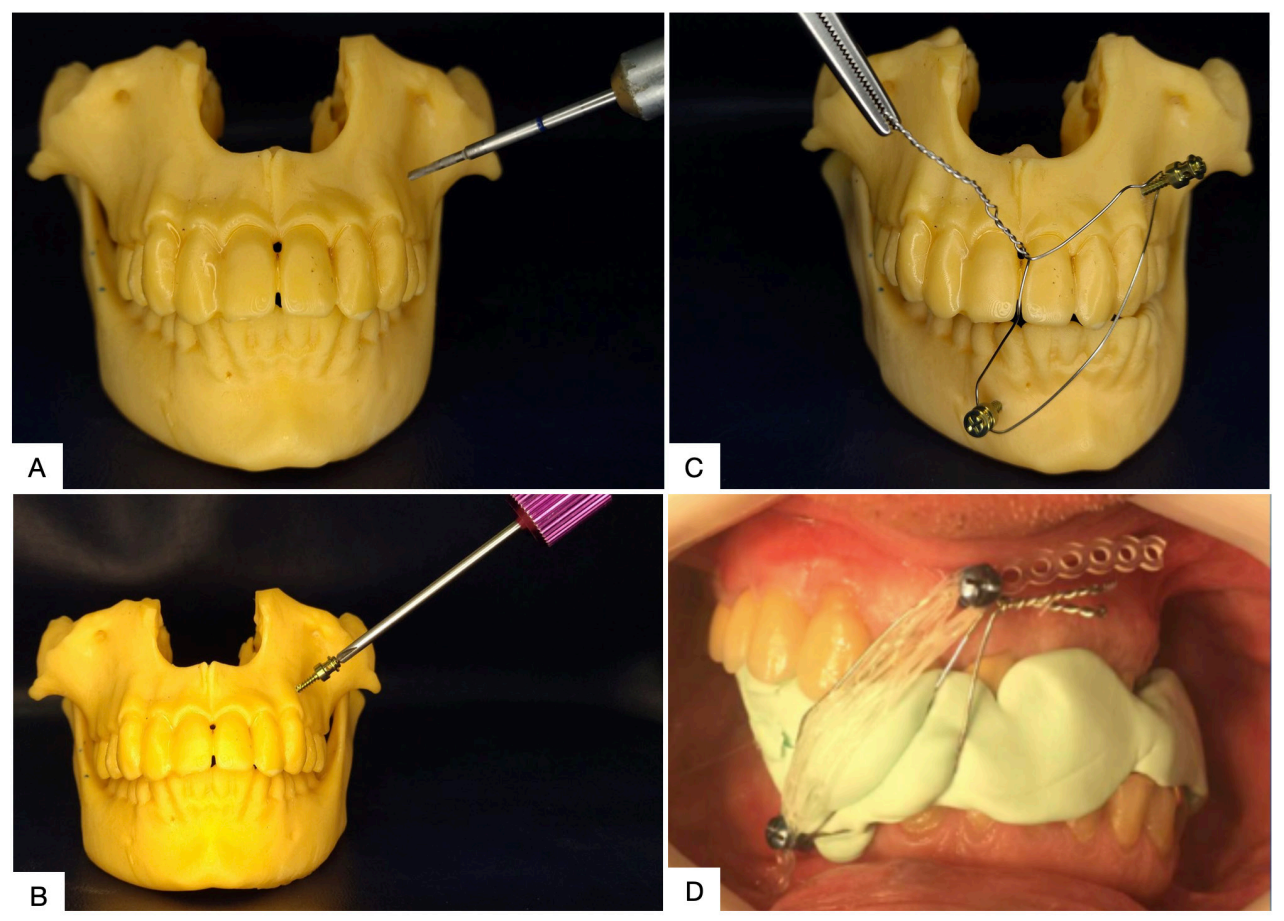
Technique for temporary mandibular subluxation (TMS) employing titanium screws. **(A)** Perforation of bone with a 1.6 mm burr; **(B)** Installation of the screws; **(C)** TMS with twisted crossed steel wires; **(D)** Application of TMS to obtain access to carotid atherosclerotic disease, employing intermaxillary molding material for additional stabilization.

A range of different techniques can be used to achieve TMS, including interdental steel wiring (1.0 mm) around the canine and/or premolar teeth (Figures 4 and 5); placement of Erich bars, Steinmann pins, or titanium miniscrews (2.0 x 10 mm), which are inserted directly into the mandible and maxilla through the oral mucosa in the vicinity of the premolars and contralateral canines and used to anchor steel wires (Figure 6); or even circummandibular wiring, in which a perforating instrument such as a Reverdin needle is inserted through the skin to loop steel wires around the mandibular body, adjacent to the periosteum and through the oral mucosa.^3^^,^^17^^,^^21^ However, with the adoption of titanium miniscrews, the last of these techniques now has fewer applications because it is more complex to perform.

The least complicated of the many different techniques is interdental steel wiring, in which the steel wires are twisted together, resulting in unilateral subluxation of the mandible (Figures 4 and 5). During these manipulations, care should be taken to avoid provoking iatrogenic displacement of the condyle to the infratemporal fossa. The correct position after subluxation of the condyle can be easily confirmed by inspection and palpation of the lateral aspect of the condyle immediately below the articular eminence.^3^

With regard to complications, from clinical studies published on SMT (investigating 217 patients), 12 reported absence of symptomatic temporomandibular dysfunction during the postoperative period;^3^^,^^15^^,^^16^^,^^19^^,^^20^^,^^22^^-^28 one did not report information on complications;^18^ and just two studies^17^^,^^21^ described patients with painful symptoms involving the temporomandibular joint (TMJ) during the postoperative period, all cases of which exhibited complete clinical resolution in a few weeks with analgesia alone. Tables S2 (available online as a supplementary file) and 4 list these studies and practical aspects related to TMS.

With the objective of protecting the TMJ from undesired luxation, Yoshino et al.^25^ described a technique for unilateral TMS employing a resin splint rapidly inserted and fixed preoperatively to guide the patient to the subluxation position. Although this technique is less invasive, it could be of limited application since it requires a specialist and advance planning, making it unlikely to be useful in emergencies.

### DSA

**Figure 7:**
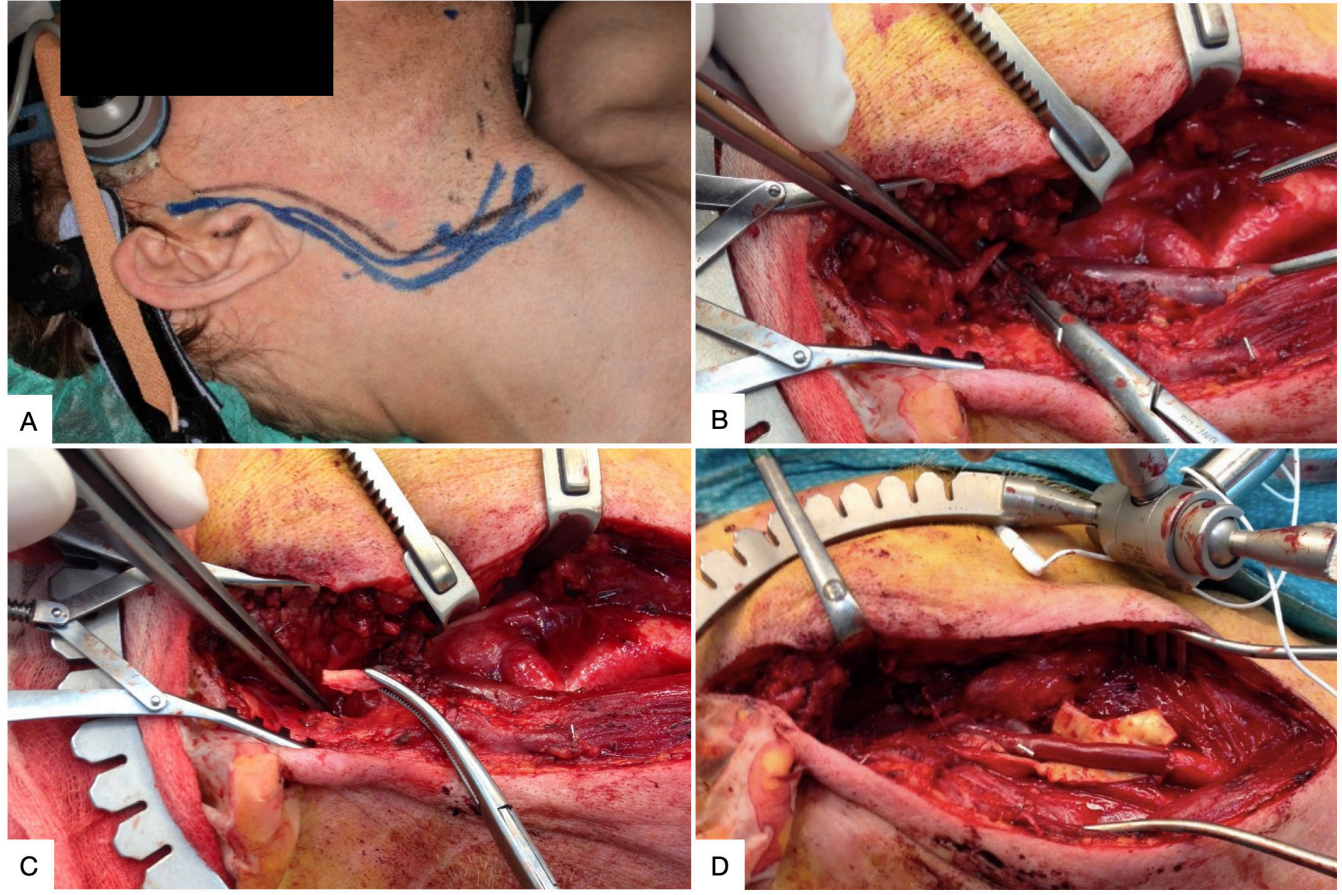
Application of sternocleidomastoid muscle retraction (SCMR), division of the digastric muscle posterior belly (DDMPB), and division of the styloid apparatus (DSA) to obtain access to a pseudoaneurysm after dissection of the internal carotid artery secondary to fibromuscular dysplasia. **(A)** Planning the cervical incision with pre-auricular extension; **(B)** Surgical access with SCMR + DDMPB and exposure of the styloid process; **(C)** Resection of the styloid process; **(D)** Reconstruction of the internal carotid artery with a venous graft.

After section or retraction of the DMPB, a further anatomic limitation is imposed by the styloid apparatus, which is made up of the styloid process, the stylohyoid, stylopharyngeus and styloglossus muscles, and the stylohyoid and stylomandibular ligaments.^5^ This region can be accessed via the infratemporal fossa or the retromandibular fossa.^5^^,^^10^ The literature recommends either that the muscles be sectioned and the styloid process resected (Figure 7) or that a styloidectomy be performed, preserving the muscle insertion points (Table S2, available online as a supplementary file).

The critical aspects of this access include the risk of neurological lesions, especially to the IX cranial nerve, which can be found medial of the styloid process and lateral of the stylopharyngeus muscle, running medially to innervate the muscles of the pharynx.^5^^,^^61^^,^^62^ In order to minimize this risk, Beretta et al.^5^ propose careful dissection of the ICA more posterior to the stylopharyngeus muscle. Additionally, the styloid process should be sectioned in the direction of the temporal bone to avoid injuries to the VII cranial nerve. Practical aspects related to DSA are shown in Table 3.

Fourth stage: MDO (for injuries above C1)

Mandibular osteotomies constitute another option for overcoming the mechanical limitations imposed by the mandibular angle and ramus. They can be performed in isolation or with rotation or temporary removal of a portion of the mandible, increasing surgical exposure. The additional exposure varies from 10 to 26 mm.^5^^,^^6^ Since MDO increase the operating time (by around 30 minutes),^40^ they are normally employed in elective surgery, such as extensive tumor resection, although there are reports of their use in trauma cases.^35^^,^^36^

**Figure 8:**
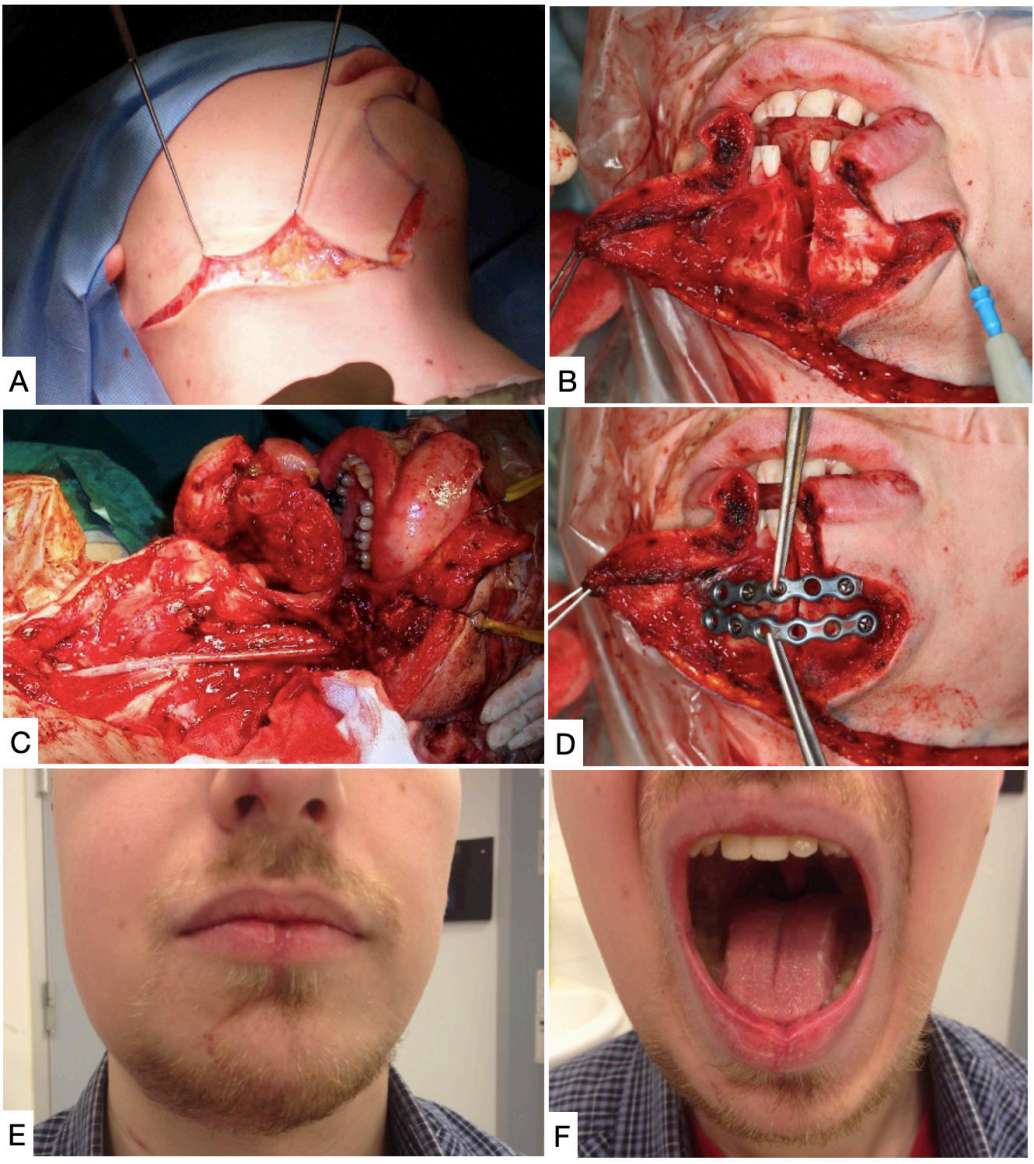
Application of the mandibular osteotomy technique for exeresis of carotid body tumor. **(A)** Cervical incision with mental extension; **(B)** Osteotomy of the mandibular symphysis; **(C)** Carotid reconstruction with a venous graft; **(D)** Fixation of the osteotomy with 2.0 mm titanium miniplates; **(E)** and **(F)** Patient in second postoperative month.

The literature describes several different MDO techniques, the most widely used of which are horizontal or vertical ramus osteotomy, subcondylar osteotomy, and osteotomies of the mandibular body, symphysis, or parasymphysis (Figure 8), or combinations of these (Tables S1 and S2, available online as a supplementary file). Irrespective of the technique employed, NTI/OEI with submental (SM) conversion/surgical AW is necessary.

Before conducting an MDO, it is recommended that titanium miniplates should be premolded and fixed and then removed afterwards. The MDO is performed and the distal segment is retracted laterally and rotated superiorly and anteriorly. During synthesis, the bone segments are fixed in their original positions. Practical aspects related to MDO are shown in Table 4.

**Table 4:** Most used mandibular osteotomy variants for access to the distal segment of the internal carotid artery or a high carotid bifurcation.

**Osteotomy**	**Advantages**	**Disadvantages**	**Complications**	**Time**
Ramus	Access can be obtained via the same incision made to expose the carotid. Exposure and sectioning are planned to avoid injuries to the facial and mandibular nerves.	Increased operating time.	Postoperative infection and malocclusion.	-
Subcondylar	Preserves the inferior and lingual alveolar nerves. Avoids toothed areas of the mandible.	Increased operating time.	Postoperative infection and malocclusion.	-
Anterolateral	Larger access.	Increased operating time.	Postoperative infection and malocclusion; more visible scarring of chin and lip; prior extraction of the tooth related to the osteotomy line; high risk of damage to inferior alveolar nerve.	-

### Proposed protocol for approach to ICA or HCB lesions

**Figure 9:**
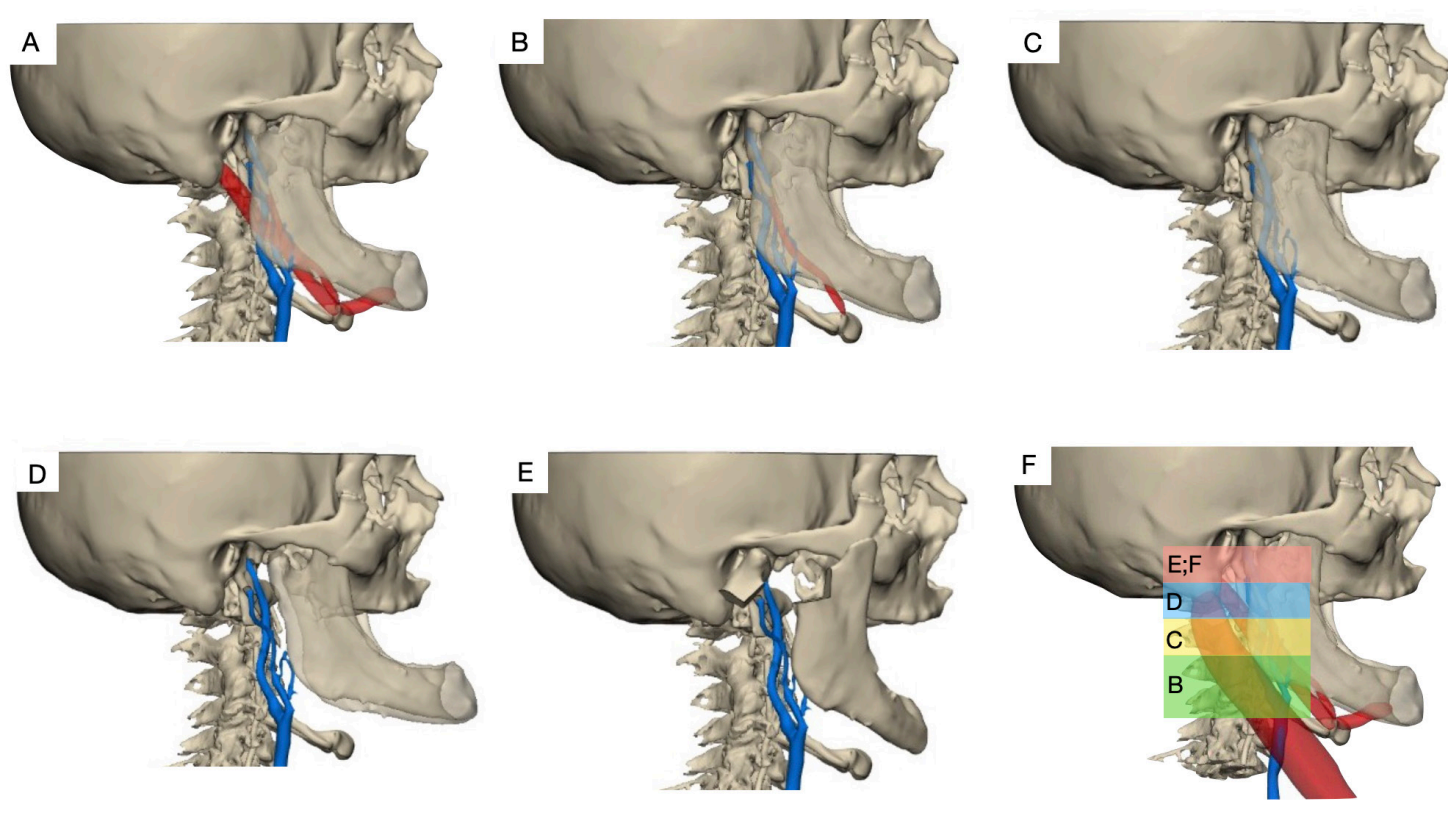
Images illustrating techniques for access to the distal segment of the internal carotid artery (ICA); planned using computed tomography images processed with Mimics® 24.0 software (Materialise, Leuven, Belgium). **(A)** Access by retraction of the sternocleidomastoid muscle (SCMR); **(B)** Access by SCMR and division of the digastric muscle posterior belly (DDMPB); **(C)** Access by SCMR + DDMPB and division of the styloid apparatus (DSA); **(D)** Access by SCMR + DDMPB + DSA and temporary mandibular subluxation (TMS); **(E)** Access by SCMR + DDMPB + DSA and mandibular osteotomy; **(F)** Illustration of levels of exposure of the ICA according to the technique employed.

**Figure 10:**
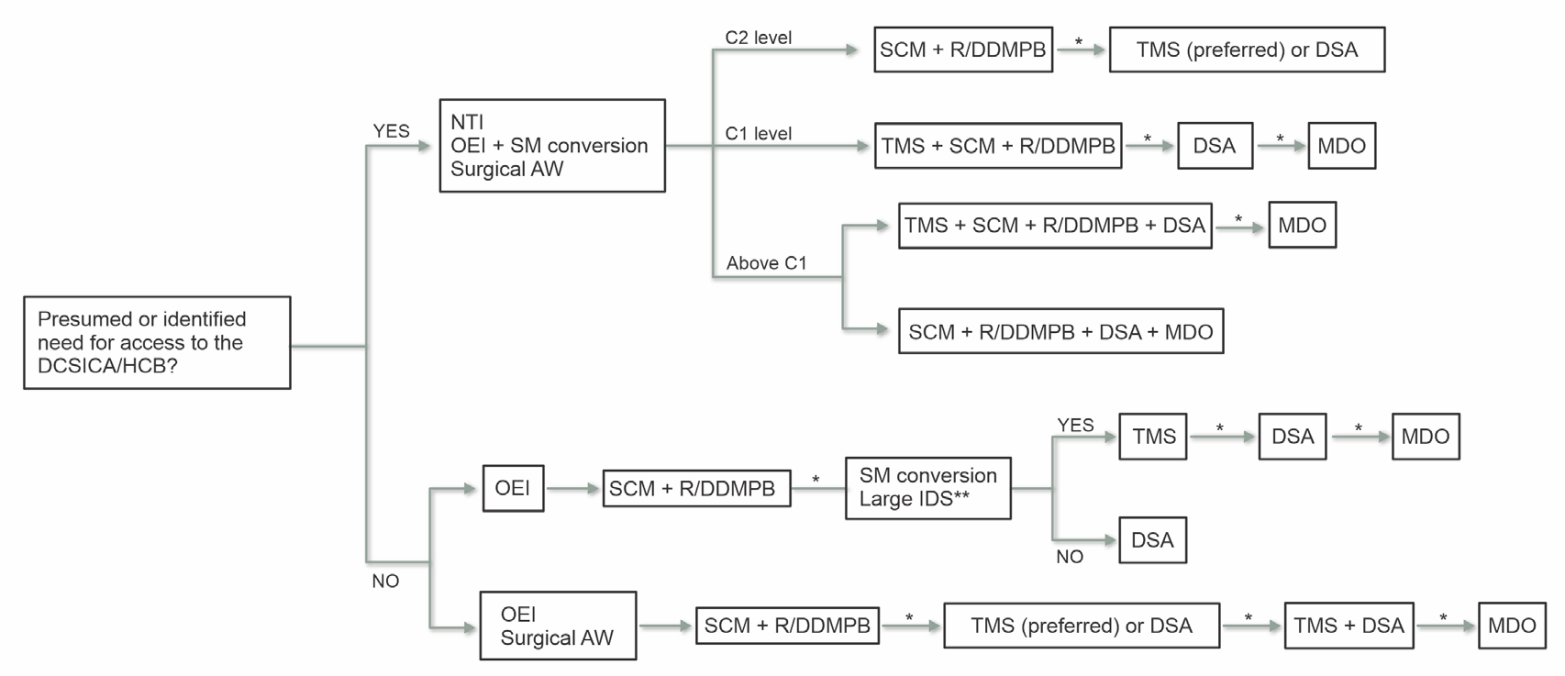
Proposed algorithm for obtaining access to lesions of the distal segment of the internal carotid artery (DCSICA) and high carotid bifurcation (HCB). DSA: division of the styloid apparatus; SCM: sternocleidomastoid; IDS: interdental spaces; NTI: nasotracheal intubation; OEI: oral endotracheal intubation; MDO: mandibular osteotomies; R/DDMPB: retraction or division of the digastric muscle posterior belly; SM: submental; TMS: temporary mandibular subluxation; AW: airway. * Need for additional exposure of the ICA identified. ** If there is sufficient space to accommodate the oral endotracheal tube.

Having reviewed the principal maneuvers described for approach to lesions of the DCSICA or an HCB, illustrated in Figure 9, it can be concluded that there is a lack of clarity with regard to the best sequence for obtaining exposure in each case. There are several factors that can help the surgeon to select the sequence of techniques, including professional expertise, especially in the case of TMS and MDO techniques, which require more specific knowledge, time available for decision-making, and hospital resources. These factors primarily affect MDO techniques, which increase the operating time considerably and require titanium miniplates and screws, in addition to the surgeon’s own subjectivity when weighing up the advantages and disadvantages of each technique. Thus, to facilitate professionals’ decision-making, we propose the following protocol (Figure 10).

Initially, the level of the lesion should be determined objectively using angiotomography in stable patients or presumptively by application of anatomic criteria in unstable patients. Once determined, the following sequence is suggested:

1. High lesions (for which the need for expanded access has been identified preoperatively): AW is recommended via NTI or OEI + SM conversion or surgical AW.
  1. Lesions at the level of C2: obtain access at the SCM + retraction or division of the DMPB (R/DDMPB) and if there is a need for additional exposure, proceed with TMS (preferable) or DSA;
  2. Lesions at the level of C1: perform TMS + access to the SCM + R/DDMPB and, if there is a need for additional exposure, proceed with DSA, followed by MDO if necessary;
  3. Lesions above C1: perform TMS + access to the SCM + R/DDMPB + DSA and, if there is a need for additional exposure, perform MDO. If TMS is not an option, obtain access at the SCM + R/DDMPB + DSA + MDO.
2. Lesions for which the need for expanded access is identified intraoperatively: analyze the AW.
  1. OEI: obtain access at the SCM + R/DDMPB and, if there is a need for additional exposure, assess the a possibility of SM conversion of OEI or whether there is sufficient interdental space to accommodate the OET. If there is, proceed with TMS, followed by DSA, or a combination of these maneuvers and MDO. If not, the only maneuver feasible is DSA;
  2. NTI or surgical AW: obtain access at the SCM + R/DDMPB and, if there is a need for additional exposure, proceed with TMS (preferable) or DSA, followed by a combination of these maneuvers and MDO.

It is worth noting that in some situations, after sufficient surgical exposure of the lesion has been obtained, ICA reflux can be controlled by inflation of balloons and catheters or with shunts which, in addition to providing hemostasis, also maintain continuous flow through the ICA.

## CONCLUSIONS

Surgical access to the distal region of the ICA or an HCB is challenging, especially in scenarios in which anatomy is distorted (hematoma, bleeding, aneurysm, tumors), increasing the difficulty and potential morbidity of the maneuvers needed. Among all of the maneuvers described, it appears reasonable that the first two steps should be to obtain access at the SCM muscle, followed by section/retraction of the DMPB. Temporary mandibular subluxation is an additional option and is preferable to DSA because of its lesser potential for morbidity. Even larger exposures can be obtained using MDO.

## Supplementary Material

Supplementary material accompanies this paper.

Table S1. Experimental studies that describe protocols for surgical access to the DSICA or a HCB.

Table S2. Clinical studies that describe protocols for surgical access to the DSICA or an HCB.

This material is available as part of the online article from https://www.scielo.br/j/jvb
