# Supplementary material for: Surgical access to the distal cervical segment of the internal carotid artery and to a high carotid bifurcation – integrative literature review and protocol proposal
Source: J Vasc Bras. 2022 Aug 8;21:e20210193. doi: 10.1590/1677-5449.202101931 (PMC9388048; doi:10.1590/1677-5449.202101931)
Supplement: Table S1. [file jvb-21-e20210193-suppl01-en.pdf]

SUPPLEMENTARY MATERIAL - TABLE S1. Experimental studies that describe protocols for surgical access to the DSICA or a HCB.

Authors	Technique	Exposure	RL/EL
Mock et al.[6]<br>(n=12)	SCMR<br>SCMR + DDMPB<br>SCMR + DDMPB + TMS<br>SCMR + DDMPB + TMS + DSA<br>SCMR + DDMPB + TMS + DSA + MDO	Superior third of C2<br>Mid third of C1 (+ 1.2 cm)<br>Superior third of C1 (+ 1.94 cm)<br>Above C1 (+ 2.26 cm)<br>Above C1 (+ 2.63 cm)	C4
Kyia et al.[7]<br>(n=13)	Deinsertion of the SCM (except posterior) + SCMR + RPDMB + DSA + MAST	DSICA	C4
Devlin et al.[8]<br>(n=5)	Deinsertion of the SCM (control)<br>Deinsertion of the SCM + TMS<br>Deinsertion of the SCM + VMRO	+ 2.8 cm<br>+ 5.5 cm (+10 cm in relation to the control)	C4
Beretta et al.[5]<br>(n=10)	SCMR<br>SCMR + DDMPB<br>SCMR + DDMPB + DSA<br>SCMR + DDMPB + DSA + MDO	2.7 cm from the CCA bifurcation<br>+ 1.4 cm<br>+ 1.5 cm<br>+ 1.0 cm	C4
Fortes et al.[9]<br>(n=30)	SCMR + DDMPB<br>SCMR + DDMPB + DSA<br>SCMR + DDMPB + DSA + TMS	3.68 cm<br>4.75 cm<br>5.52 cm	C4
Izci et al.[10] (n=4)	SCMR + DDMPB + DSA	6.8 cm of the length of the cervical ICA in the retromandibular fossa	C4
Batzdorf & Gregorius[11] (n=5)	SCMR + RPDMB + MBO + HMRO	DSICA	C4
Tanjararak et al.[12]<br>(n=10)	TC/submandibular: SCMR + RPDMB<br>TC/transparotid: SCMR + DDMPB + DSML<br>TC/transmandibular: SCMR + RPDMB + MSO	Inferior and medial portion of the PPS (3.69 cm of the ICA<br>Inferior and medial portion of the PPS (4.595 cm of the ICA<br>All portions of the PPS (7 cm of the ICA	C4
Yalvac at al.[13]<br>(n=2)	SCMR<br>SCMR + DDMPB	+ 2.0 cm distal	C4
Ammirati et al.[14]<br>(n=10)	SCMR + DDMPB + MSO<br>SCMR + DDMPB + MSO + DSA	Pre-styloid segment of the parapharyngeal space<br>Post-styloid segment of the parapharyngeal space	C4

CCA = common carotid artery; = = internal carotid artery; HCB = high carotid bifurcation; DSA = division of the styloid apparatus; DSML = division of the stylomandibular ligament ; DDMPB = division of the digastric muscle posterior belly ; SCM = sternocleidomastoid muscle; PPS = parapharyngeal space; RL = recommendation level; MAST = mastoidectomy; EL = evidence level; MBO = mandibular body osteotomy; HMRO = horizontal mandibular ramus osteotomy ; MDO = mandibular osteotomy; MSO = mandibular symphysis osteotomy; VMRO = vertical mandibular ramus osteotomy ; SCMR = sternocleidomastoid retraction; RPDMB = retraction of the digastric muscle posterior belly ; DSICA = distal segment of the internal carotid artery; TMS = temporary mandibular subluxation; TC = transcervical.

SUPPLEMENTARY MATERIAL - TABLE S2. Clinical studies that describe protocols for surgical access to the DSICA or an HCB.

Author	Indications	Technique	Level of lesion /exposure	RL/EL
Fisher et al.[3] (n=24)	Atheromatous plaques, trauma, neoplasm, aneurysms, fistula	TMS + SCMR + DDMPB + DSA	+ 1 to 2 cm distal	C4
Mock et al.[6] (n=3)	ICA stenosis<br>Aneurysm<br>Trauma (CWW)	SCMR + DDMPB<br>TMS + SCMR + DDMPB<br>TMS + SCMR + DDMPB + MAST	3 cm distal of the BCCA;<br>area between the mandibular angle and the MP;<br>1 cm from the carotid canal	C4
Goldsmith et al.[15] (n=3)	Trauma (CWW)	TMS + SCMR + RPDMB + retraction of the styloid muscle	1 cm proximal of the base of the skull	C4
Cantore et al.[16] (n=34)	Malformations, fibromuscular dysplasia, aneurysm, atheromatous plaques	TMS + SCMR + DDMPB + DSA	DSICA	C4
Dossa et al.[17] (n=14)	Atheromatous plaques, neoplasm, trauma, pseudoaneurysm	TMS + SCMR + DDMPB + DSA	Above the Blaisdell line	C4
Moreau et al.[18] (n=35)	Aneurysm	SCMR + DDMPB + DSA<br>Deinsertion of the SCM (5 cases)<br>TMS (2 cases)	DSICA	C4
Frim et al.[19] (n=6)	Atheromatous plaques	TMS*	Level of C1	C4
Cartier et al.[20] (n=1)	Recurrent stenosis	TMS*	DSICA	C4
Simonian et al.[21] (n=10)	Stenosis and aneurysm	TMS + SCMR + DDMPB + DSA	Above the Blaisdell line	C4
Mccabe et al.[22] (n=1)	Atheromatous plaques	TMS*	DSICA	C4
Puggioni et al.[23] (n=1)	Carotid body tumor	TMS + SCMR + DDMPB	3.0 to 3.5 cm from the base of the skull	C4
Jaspers et al.[24] (n=4)	Atheromatous plaques	TMS*	DSICA	C4
Yoshino et al.[25] (n=28)	Atheromatous plaques	TMS + SCMR + RPDMB	Level of the midpoint of C2	C4
Capoccia et al.[26] (n=43)	Atheromatous plaques, stenosis, pseudoaneurysm, neoplasm	TMS + SCMR + DDMPB	Above the Blaisdell line	C4
Santos et al.[27] (n=1)	Atheromatous plaques	TMS + SCMR + DDMPB	High carotid bifurcation	C4
Ifle et al.[28] (n=1)	Aneurysm	TMS + SCMR + DDMPB	4 cm from the base of the skull	C4
Rhee et al.[29] (n=2)	Aneurysm	TMS*	7 cm above the carotid bulb;<br>6 cm above the CCA bifurcation	C4

Author	Indications	Technique	Level of lesion /exposure	RL/EL
Shaha et al.[30] (n=2)	Trauma (CWW)	SECM + DDMPB + DSA + MAST	3 cm above the CCA bifurcation	C4
Sasaki et al.[31] (n=20)	Atheromatous plaques	Longitudinal section and SCMR	Level of the C1 vertebral body	C4
Hans et al.[32] (n=14)	Atheromatous plaques	SCMR + RPDMB	Zone II	C4
Malikov et al.[33] (n=13)	Aneurysm	SCMR + DDMPB + DSA + TMS	Level with the base of the skull	C4
Smith et al.[34] (n=3)	Carotid body tumor	SCMR + VMRO + MPSO	Level with the base of the skull	C4
Dichtel et al.[35] (n=1)	Trauma (CWW)	SCMR + DDMPB + MPSO	Level with the base of the skull	C4
Larsen & William[36] (n=2)	Trauma (CWW)<br>Atheromatous plaques	SCMR + EST + VMRO<br>SCMR + VMRO	5 mm from the base of the skull;<br>high carotid bifurcation	C4
Ktenidis et al.[37] (n=1)	Aneurysm	SCMR + MPSO + SCO + coronoidectomy	Above the Blaisdell line	C4
Balagura et al.[38] (n=2)	Stenosis, ulceration<br>Aneurysm	SCMR + MPSO<br>SCMR+ DDMPB + MPSO + HMRO	Level of C1-C2	C4
Buckley et al.[39] (n=15)	Atheromatous plaques, aneurysm, neoplasm	MPSO + SCO	DSICA	C4
Kumins et al.[40] (n=8)	Atheromatous plaques	SCMR + DDMPB + VMRO	1.5 cm from the base of the skull	C4
Nelson et al.[41] (n=16)	Information not provided	SMRO or VMRO	Zone III	C4
Schlieve et al.[42] (n=17)	Aneurysm, neoplasms, stenosis	MPSO + SCO	Level of C1-C2, at the base of the skull, and in the parapharyngeal space	C4
Vikatmaa et al.[43] (n=5)	Aneurysm and neoplasms	MSO + lateral luxation of the mandible*	DSICA	C4
Hafner et al.[44] (n=1)	Aneurysm	SCMR + DSA + MBO	Projection of C2 and C3 (base of the skull)	C4
Valentini et al.[45] (n=1)	Stenosis	SCMR + VMRO	DSICA	C4
Davis et al.[46] (n=1)	Aneurysm	SCMR + DDMPB + DSA + MPSO + VMRO	1 cm from the base of the skull	C4
Kawanishi et al.[47] (n=1)	Aneurysm	SCMR + DDM + DSA + VMRO	Level of C2	C4
Farhat-Sabet at al.[48] (n=4)	Atheromatous plaques	SCMR + RPDMB	C2-C3 intervertebral level;<br>C2 vertebral level	C4
Ben Jmaà et al.[49] (n=1)	Aneurysm	SCMR + DDM	2 cm above the CCA bifurcation (close to the base of the skull)	C4
Bakoyiannis et al.[50] (n=9)	Aneurysm	SCMR + RPDMB or DDMPB + DSA	Below and above the Blaisdell line	C4
Pennel et al.[51] (n=1)	Aneurysm	DSCM + DDM + DSA	1.5 cm distal of the origin of the ICA	C4

Comentado [R1]: AUTOR: Favor informe o significado dessa sigla.

Comentado [R2]: AUTOR: Favor informe o significado dessa sigla.

Author	Indications	Technique	Level of lesion /exposure	RL/EL
Sundt et al.[52] (n=19)	Aneurysm	SCMR + DDMPB + DSA	Level de C2; distal of the styloid process; level with the base of the skull	C4
Awasthi & Smith[4] (n=1)	Stenosis	SCMR + DDMPB + DSA<br>Styloid partially resected	Level of C1-C2	C4
Sandman et al.[53] (n=31)	Aneurysm<br>Stenosis	SCMR + DDM + DSA + MAST partial	High ICA lesions	C4
Mendez-Sosa et al.[54] (n=1)	Aneurysm	SCMR + Thompson retractor	1 cm below the base of the skull	C4
Vang et al.[55] (n=2)	Atheromatous plaques	TMS*	Zones II and III	C4
Kondo et al.[56] (n=34)	Atheromatous plaques	SCMR + RPDMB	Above C2	C4
Coll et al.[57] (n=43)	Atheromatous plaques	SCMR + DDMPB + DSA + retractor	Level of C1	C4

CCA = common carotid artery; ICA= internal carotid artery; BCCA = bifurcation of the common carotid artery; HCB= high carotid bifurcation; DSA = division of the styloid apparatus; DSCM = division of the sternocleidomastoid; DDM = division of the digastric muscle; DDMPB = division of the digastric muscle posterior belly ; SCM = sternocleidomastoid muscle; CWW = cold weapon wound; GSW = gunshot wound; RL = recommendation level; MAST = mastoidectomy; EL = evidence level; MBO = mandibular body osteotomy ; HMRO = horizontal mandibular ramus osteotomy; SCO = subcondylar osteotomy; MPSO = mandibular parasymphysis osteotomy; MSO = mandibular symphysis osteotomy ; SMRO = sagittal mandibular ramus osteotomy ; VMRO= vertical mandibular ramus osteotomy; MP= mastoid process; SCMR = sternocleidomastoid retraction; RPDMB= retraction of the digastric muscle posterior belly ; DSICA = distal segment of the internal carotid artery; TMS = temporary mandibular subluxation; TC = transcervical.

* Does not describe the stages of surgical access.
