# Supplementary material for: Surgical access to the distal cervical segment of the internal carotid artery and to a high carotid bifurcation – integrative literature review and protocol proposal
Source: J Vasc Bras. 2022 Aug 8;21:e20210193. doi: 10.1590/1677-5449.202101931 (PMC9388048; doi:10.1590/1677-5449.202101931)
Supplement: Tabela S1. [file jvb-21-e20210193-suppl01.pdf]

MATERIAL SUPLEMENTAR - TABELA S1. Estudos experimentais que descrevem protocolos de acesso cirúrgico ao SDACI ou à BCA.

Autor	Técnica	Exposição	GR/NE
Mock et al.[6]<br>(n=12)	RECM<br>RECM + DVPD<br>RECM + DVPD + SMT<br>RECM + DVPD + SMT + DAE<br>RECM + DVPD + SMT + DAE + OMD	Terço superior de C2<br>Terço médio de C1 (+ 1,2 cm)<br>Terço superior de C1 (+ 1,94 cm)<br>Acima de C1 (+ 2,26 cm)<br>Acima de C1 (+ 2,63 cm)	C4
Kyia et al.[7]<br>(n=13)	Desinserção do ECM (exceto posterior) + RECM + RVPD + DAE + MAST	SDACI	C4
Devlin et al.[8]<br>(n=5)	Desinserção do ECM (controle)<br>Desinserção do ECM + SMT<br>Desinserção do ECM + OVRM	+ 2,8 cm<br>+ 5,5 cm (+10 cm em relação ao controle)	C4
Beretta et al.[5]<br>(n=10)	RECM<br>RECM + DVPD<br>RECM + DVPD + DAE<br>RECM + DVPD + DAE + OMD	2,7 cm da bifurcação da ACC<br>+ 1,4 cm<br>+ 1,5 cm<br>+ 1,0 cm	C4
Fortes et al.[9]<br>(n=30)	RECM + DVPD<br>RECM + DVPD + DAE<br>RECM + DVPD + DAE + SMT	3,68 cm<br>4,75 cm<br>5,52 cm	C4
Izci et al.[10] (n=4)	RECM + DVPD + DAE	6,8 cm de comprimento da ACI cervical na fossa retromandibular	C4
Batzdorf & Gregorius[11] (n=5)	RECM + RVPD + OCM + OHRM	SDACI	C4
Tanjararak et al.[12]<br>(n=10)	TC/submandibular: RECM + RVPD<br>TC/transparotídeo: RECM + DVPD + DLSM<br>TC/transmandibular: RECM + RVPD + OSM	Porção inferior e média do EPF (3,69 cm da ACI)<br>Porção inferior e média do EPF (4,595 cm da ACI)<br>Todas as porções do EPF (7 cm da ACI)	C4
Yalvac at al.[13]<br>(n=2)	RECM<br>RECM + DVPD	+ 2,0 cm distal	C4
Ammirati et al.[14]<br>(n=10)	RECM + DVPD + OSM<br>RECM + DVPD + OSM + DAE	Segmento pré-estiloide do espaço parafaríngeo<br>Segmento pós-estiloide do espaço parafaríngeo	C4

ACC = artéria carótida comum; ACI = artéria carótida interna; BCA = bifurcação carotídea alta; DAE = divisão do aparto estiloide; DLSM = divisão do ligamento estilomandibular; DVPD = divisão do ventre posterior do músculo digástrico; ECM = músculo esternocleidomastóideo; EPF = espaço parafaríngeo; GR = grau de recomendação; MAST = mastoidectomia; NE = nível de evidência; OCM = osteotomia de corpo mandibular; OHRM = osteotomia horizontal de ramo mandibular; OMD = osteotomia mandibular; OSM = osteotomia de sínfise mandibular; OVRM = osteotomia vertical de ramo mandibular; RECM = retração do esternocleidomastóideo; RVPD = retração do ventre posterior do músculo digástrico; SDACI = segmento distal da artéria carótida interna; SMT = subluxação mandibular temporária; TC = transcervical.

MATERIAL SUPLEMENTAR - TABELA S2. Estudos clínicos que descrevem protocolos de acesso cirúrgico ao SDACI ou à BCA.

Autor	Indicações	Técnica	Nível da lesão/exposição	GR/NE
Fisher et al.[3] (n=24)	Placa de ateroma, trauma, neoplasia, aneurismas, fístula	SMT + RECM + DVPD + DAE	+ 1 a 2 cm distal	C4
Mock et al.[6] (n=3)	Estenose de ACI<br>Aneurisma<br>Trauma (FAB)	RECM + DVPD<br>SMT + RECM + DVPD<br>SMT + RECM + DVPD + MAST	3 cm distal à BACC;<br>área entre o ângulo mandibular e o PM;<br>1 cm do canal carotídeo	C4
Goldsmith et al.[15] (n=3)	Trauma (FAB)	SMT + RECM + RVPD + retração do músculo estiloide	1 cm proximal à base do crânio	C4
Cantore et al.[16] (n=34)	Malformações, displasia fibromuscular, aneurisma, placa de ateroma	SMT + RECM + DVPD + DAE	SDACI	C4
Dossa et al.[17] (n=14)	Placa de ateroma, neoplasia, trauma, pseudoaneurisma	SMT + RECM + DVPD + DAE	Acima da linha de Blaisdell	C4
Moreau et al.[18] (n=35)	Aneurisma	REMC + DVPD + DAE<br>Desinserção do ECM (5 casos)<br>SMT (2 casos)	SDACI	C4
Frim et al.[19] (n=6)	Placa de ateroma	SMT*	Nível de C1	C4
Cartier et al.[20] (n=1)	Estenoses recorrentes	SMT*	SDACI	C4
Simonian et al.[21] (n=10)	Estenoses e aneurisma	SMT + RECM + DVPD + DAE	Acima da linha de Blaisdell	C4
Mccabe et al.[22] (n=1)	Placa de ateroma	SMT*	SDACI	C4
Puggioni et al.[23] (n=1)	Tumor de corpo carotídeo	SMT + RECM + DVPD	3,0 a 3,5 cm da base craniana	C4
Jaspers et al.[24] (n=4)	Placa de ateroma	SMT*	SDACI	C4
Yoshino et al.[25] (n=28)	Placa de ateroma	SMT + RECM + RVPD	Nível do ponto médio de C2	C4
Capoccia et al.[26] (n=43)	Placa de ateroma, estenoses, pseudoaneurisma, neoplasia	SMT + RECM + DVPD	Acima da linha de Blaisdell	C4
Santos et al.[27] (n=1)	Placa de ateroma	SMT + RECM + DVPD	Bifurcação carotídea alta	C4
Ifle et al.[28] (n=1)	Aneurisma	SMT + RECM + DVPD	4 cm da base craniana	C4
Rhee et al.[29] (n=2)	Aneurisma	SMT*	7 cm acima do bulbo carotídeo;<br>6 cm acima da bifurcação da ACC	C4
Shaha et al.[30] (n=2)	Trauma (FAF)	SECM + DVPD + DAE + MAST	3 cm acima da bifurcação da ACC	C4
Sasaki et al.[31] (n=20)	Placa de ateroma	Secção longitudinal e RECM	Nível do corpo vertebral de C1	C4

Autor	Indicações	Técnica	Nível da lesão/exposição	GR/NE
Hans et al.[32] (n=14)	Placa de ateroma	REMC + RVPD	Zona II	C4
Malikov et al.[33] (n=13)	Aneurisma	RECM + DVPD + DAE + SMT	Nível da base craniana	C4
Smith et al.[34] (n=3)	Tumor de corpo carotídeo	RECM + OVRM + OPSM	Nível da base craniana	C4
Dichtel et al.[35] (n=1)	Trauma (FAF)	RECM + DVPD + OPSM	Nível da base craniana	C4
Larsen & William[36] (n=2)	Trauma (FAF)<br>Placa de ateroma	RECM + EST + OVRM<br>RECM + OVRM	5 mm da base craniana;<br>bifurcação carotídea alta	C4
Ktenidis et al.[37] (n=1)	Aneurisma	RECM + OPSM + OSC + coronoidectomia	Acima da linha de Blaisdell	C4
Balagura et al.[38] (n=2)	Estenose, ulceração<br>Aneurisma	RECM + OPSM<br>RECM+ DVPD + OPSM + OHRM	Nível de C1-C2	C4
Buckley et al.[39] (n=15)	Placa de ateroma, aneurisma, neoplasia	OPSM + OSC	SDACI	C4
Kumins et al.[40] (n=8)	Placa de ateroma	RECM + DVPD + OVRM	1,5 cm da base craniana	C4
Nelson et al.[41] (n=16)	Não informado	OSRM ou OVRM	Zona III	C4
Schlieve et al.[42] (n=17)	Aneurisma, neoplasias, estenose	OPSM + OSC	Nível de C1-C2, da base craniana e no espaço parafaríngeo	C4
Vikatmaa et al.[43] (n=5)	Aneurisma e neoplasias	OSM + luxação lateral da mandíbula*	SDACI	C4
Hafner et al.[44] (n=1)	Aneurisma	REMC + DAE + OCM	Projeção de C2 e C3 (base craniana)	C4
Valentini et al.[45] (n=1)	Estenose	REMC + OVRM	SDACI	C4
Davis et al.[46] (n=1)	Aneurisma	REMC + DVPD + DAE + OPSM + OVRM	1 cm da base craniana	C4
Kawanishi et al.[47] (n=1)	Aneurisma	REMC + DMD + DAE + OVRM	Nível de C2	C4
Farhat-Sabet at al.[48] (n=4)	Placa de ateroma	REMC + RVPD	Nível intervertebral entre C2-C3;<br>nível vertebral de C2	C4
Ben Jmaà et al.[49] (n=1)	Aneurisma	REMC + DMD	2 cm acima da bifurcação da ACC<br>(próximo à base craniana)	C4
Bakoyiannis et al.[50] (n=9)	Aneurisma	REMC + RVPD ou DVPD + DAE	Abaixo e acima da linha de Blaisdell	C4
Pennel et al.[51] (n=1)	Aneurisma	DECM + DMD + DAE	1,5 cm distal à origem da ACI	C4
Sundt et al.[52] (n=19)	Aneurisma	REMC + DVPD + DAE	Nível de C2; distal ao processo estiloide;<br>nível da base craniana	C4
Awasthi & Smith[4]	Estenose	REMC + DVPD + DAE	Nível de C1-C2	C4

Autor	Indicações	Técnica	Nível da lesão/exposição	GR/NE
(n=1)		Estiloide parcialmente ressecado		
Sandman et al.[53] (n=31)	Aneurisma<br>Estenose	REMC + DMD + DAE + MAST parcial	Lesões altas da ACI	C4
Mendez-Sosa et al.[54] (n=1)	Aneurisma	RECM + retrator Thompson	1 cm abaixo da base craniana	C4
Vang et al.[55] (n=2)	Placa de ateroma	SMT*	Zona II e III	C4
Kondo et al.[56] (n=34)	Placa de ateroma	RECM + RVPD	Acima de C2	C4
Coll et al.[57] (n=43)	Placa de ateroma	RECM + DVPD + DAE + retrator	Nível de C1	C4

ACC = artéria carótida comum; ACI = artéria carótida interna; BACC = bifurcação da artéria carótida comum; BCA = bifurcação carotídea alta; DAE = divisão do aparto estiloide; DECM = divisão do esternocleidomastóideo; DMD = divisão do músculo digástrico; DVPD = divisão do ventre posterior do músculo digástrico; ECM = músculo esternocleidomastóideo; FAB = ferimento por arma branca; FAF = ferimento por arma de fogo; GR = grau de recomendação; MAST = mastoidectomia; NE = nível de evidência; OCM = osteotomia de corpo mandibular; OHRM = osteotomia horizontal de ramo mandibular; OSC = osteotomia subcondilar; OPSM = osteotomia de parassínfise mandibular; OSM = osteotomia de sínfise mandibular; OSRM = osteotomia sagital de ramo mandibular; OVRM = osteotomia vertical de ramo mandibular; PM = processo mastóideo; RECM = retração do esternocleidomastóideo; RVPD = retração do ventre posterior do músculo digástrico; SDACI = segmento distal da artéria carótida interna; SMT = subluxação mandibular temporária; TC = transcervical.

* Não descreve as etapas do acesso cirúrgico.
