# Supplementary material for: Surgical access to the distal cervical segment of the internal carotid artery and to a high carotid bifurcation – integrative literature review and protocol proposal
Source: J Vasc Bras. 2022 Aug 8;21:e20210193. doi: 10.1590/1677-5449.202101931 (PMC9388048; doi:10.1590/1677-5449.202101931)
Supplement: Translation

# Translation: Acesso cirúrgico ao segmento cervical distal da artéria carótida interna e à bifurcação carotídea alta – revisão integrativa da literatura e proposta de protocolo

**DOI:** 10.1590/1677-5449.202101932

**Authors:** Nicolau Conte, Thais Tapajós Gonçalves, Clarina Louis, Jonas Ikikame, Adenauer Marinho de Oliveira Góes Junior

**Affiliations:** 1 Centro Universitário do Estado do Pará – CESUPA, Belém, PA, Brasil.; 2 Universidade Federal do Pará – UFPA, Belém, PA, Brasil.

**Keywords:** artéria carótida interna, doenças das artérias carótidas, osteotomia mandibular, articulação temporomandibular

## Abstract

Diversas manobras já foram descritas para o acesso ao segmento distal cervical da artéria carótida interna ou à bifurcação carotídea alta; entretanto, há divergências na sistematização dessas técnicas. O objetivo deste estudo é revisar as técnicas descritas e propor um protocolo prático que auxilie na seleção da técnica mais adequada para cada caso. Para isso, foi realizada uma busca nas bases de dados PubMed Central, Biblioteca Virtual em Saúde e SciELO por artigos sobre o tema, em língua inglesa ou portuguesa, publicados entre os anos de 1980 e 2021. Entre as manobras descritas, parece razoável que as duas etapas iniciais sejam a abordagem ao músculo esternocleidomastóideo, seguida pela secção/retração do ventre posterior do músculo digástrico. Caso necessário, a subluxação mandibular temporária unilateral é um recurso adicional e preferível à divisão do aparato estiloide, devido ao menor potencial de morbidade. Exposições ainda mais amplas podem ser obtidas com as osteotomias mandibulares.

## INTRODUÇÃO

Em virtude das limitações anatômicas de acesso e do risco aumentado de lesões vasculares e neurológicas, a exposição cirúrgica da bifurcação carotídea alta (BCA) ou do segmento distal da artéria carótida interna (SDACI) cervical é desafiadora. A dificuldade pode ser ainda maior após traumatismos, devido a hemorragia profusa e/ou hematoma pulsátil/expansivo, nos quais o acesso rápido é relevante para o prognóstico^1^^,^^2^.

Intervenções no SDACI ou na BCA podem ser realizadas por via endovascular e/ou cirurgia aberta. Ocasionalmente, o procedimento não é realizado sobre o SDACI, mas a exposição dele é necessária. As indicações mais frequentes incluem aneurismas, neoplasias, estenose aterosclerótica e traumatismos na zona III do pescoço^3^. Independentemente da indicação, a manutenção da perviedade da artéria carótida interna (ACI) é sempre preferível, pois reduz o risco de défices neurológicos. No entanto, as limitações de acesso ao SDACI ou à BCA aumentam substancialmente o número de ligaduras da ACI^1^^,^^2^.

Para reduzir as dificuldades e os riscos envolvidos, várias manobras para a exposição do SDACI ou da BCA foram descritas. As abordagens ântero-laterais são as mais frequentes, sendo representadas pela secção/retração do músculo esternocleidomastóideo (ECM) e do ventre posterior do músculo digástrico (VPD), pela divisão do aparato estiloide (DAE), pela subluxação mandibular e por osteotomias^3^^,^^4^. Já as abordagens póstero-laterais, como a mastoidectomia, são menos comuns e normalmente reservadas para quando há envolvimento da porção petrosa da ACI^5^.

Embora alguns estudos clínicos^3^^,^^6^ e experimentais^6^^-^14 tenham proposto a utilização de uma sequência progressiva dessas técnicas de acordo com a necessidade de exposição adicional do SDACI ou da BCA, há divergências na sequência desses procedimentos. Além disso, esses estudos não enfatizam aspectos práticos relevantes, como o tempo para a tomada de decisão, recursos hospitalares necessários e a *expertise* do cirurgião.

Infelizmente, nenhuma das manobras previamente descritas para exposição cirúrgica do SDACI ou da BCA é isenta de potenciais complicações, e a falta de conhecimento por parte dos profissionais que as executam aumenta a morbidade dos procedimentos. O objetivo deste estudo é revisar as técnicas previamente descritas, reunindo-as em um único artigo e propondo um protocolo prático que auxilie na escolha da técnica mais adequada para cada caso.

## MÉTODOS

Realizou-se uma revisão integrativa da literatura a partir de estudos publicados nas bases de dados PubMed Central, SciELO e Biblioteca Virtual em Saúde. Foram selecionados os seguintes descritores: *internal carotid artery*, *distal, cervical*, *extracranial*, *carotid bifurcation*, *high*, *open surgery*, *open approach*, *exposure*, *access*, *subluxation*, *luxation*, *osteotomy*, *mandibular*, *styloid* e *digastric*. Esses termos foram adaptados ao idioma da base de dados consultada e combinados entre si utilizando operadores booleanos para o refinamento da pesquisa.

A seleção dos estudos foi realizada de acordo com os seguintes critérios de inclusão: 1) estudos clínicos e experimentais que descrevessem a(s) técnica(s) utilizada(s) para exposição carotídea; 2) lesões localizadas no SDACI ou na BCA; 3) publicações no período de janeiro de 1980 a setembro de 2021; 4) trabalhos em língua inglesa ou portuguesa. Foram excluídas publicações que 1) utilizassem abordagem endovascular ou híbrida; 2) necessitassem de exposição da porção petrosa da ACI; 3) descrevessem abordagens exclusivamente pela via póstero-lateral; ou 4) não estivessem disponíveis na íntegra.

Os artigos não selecionados para tabulação dos dados foram utilizados como referência para discussão. Além disso, foi estabelecido contato com especialistas nacionais e internacionais, solicitando o envio de imagens de casos reais, materiais previamente apresentados/publicados e a autorização para ilustração neste estudo.

## RESULTADOS

A busca inicial utilizando os descritores selecionados gerou 3.162 artigos (Figura 1). Em seguida, foram retirados os artigos duplicados e, posteriormente, aplicados os critérios de inclusão e exclusão em todas as etapas analíticas. O primeiro momento consistiu na leitura dos títulos e resumos, resultando em 80 artigos; o segundo momento consistiu na leitura dos artigos que estavam disponíveis na íntegra, resultando na inclusão de 57 artigos neste estudo. Desses, nove eram experimentais^7^^-^14, 47 eram estudos clínicos (relato e série de casos)^3^^,^^15^^-^57 e um^6^ era estudo clínico e experimental. A amostra total incluiu 101 espécimes (artérias carótidas dissecadas em cadáver) e 480 pacientes (Tabelas S1 e S2, disponíveis como arquivo *on-line* suplementar), sendo as principais indicações de exposição do SDACI ou da BCA representadas por estenoses por ateromatose e aneurismas (Tabela S2, disponível como arquivo *on-line* suplementar).

**Figura 1:** Fluxograma de seleção dos artigos. ACI: artéria carótida interna.

## DISCUSSÃO

### Anatomia cirúrgica e variações anatômicas

O segmento cervical da ACI inicia ao nível da bifurcação da artéria carótida comum (ACC) e termina ao nível do forame carotídeo. Essa bifurcação normalmente ocorre ao nível de C3/C4, posteriormente ao bordo superior da cartilagem tireoide^58^. Entretanto, variações anatômicas significativas foram descritas, incluindo os níveis de T4 e Cl^59^. Por convenção, a bifurcação carotídea é considerada “alta” quando ocorre cranialmente à C3/C4, acima do osso hioide/cartilagem tireoide^58^^,^^60^ ou acima da linha de Blaisdell (linha projetada entre o ápice do processo mastoide e o ângulo da mandíbula)^27^ e “baixa” quando ocorre inferiormente a essas referências anatômicas^58^^,^^60^.

Após a bifurcação da ACC, a ACI tem curso ascendente dentro da bainha carotídea, posteromedial à veia jugular interna e ao nervo vago, até o canal carotídeo no osso temporal^61^^,^^62^. Ao longo desse trajeto, existem variações no curso do vaso, permitindo a divisão em quatro categorias: *straight* (tipo 1), *curved* (tipo 2), *kinking* (tipo 3) e *loop* (tipo 4)^60^.

Para o planejamento cirúrgico, é relevante a divisão da porção cervical da ACI em segmentos proximal e distal. Para isso, diversos marcos anatômicos foram descritos, como a altura do cruzamento do nervo hipoglosso sobre a ACI, a linha de Blaisdell e o VPD^3^^,^^5^^,^^9^. O segmento proximal estende-se da bifurcação da ACC até esses marcos, e o segmento distal compreende a porção da ACI entre essas estruturas e a base do crânio^61^^,^^62^. As estruturas anatômicas relacionadas à abordagem da ACI encontram-se na Tabela 1.

**Tabela 1:** Principais estruturas encontradas na topografia do acesso à artéria carótida interna^61^^,^^62^.

**Estrutura**	**Marcos anatômicos**	**Repercussões após lesão**
Nervo V_3_ (trigêmeo/ramo mandibular)	Emerge do crânio a partir do forame oval. Trajeto inferior associado à artéria alveolar inferior e medial ao ramo mandibular. Segue profundo ao músculo pterigoideo lateral e depois segue entre o ligamento esfenomandibular e o ramo da mandíbula até o forame mandibular.	Parestesia dos elementos dentários da mandíbula, da região do mento e do lábio inferior.
Nervo VII (facial)	Emerge da base do crânio a partir do forame estilomastóideo. Percorre na mesma profundidade ou ligeiramente mais profundo do que a borda superior do ventre posterior do músculo digástrico. É acompanhado pela artéria estilomastóidea. Relação com a fissura timpanomastóidea, que fica de 7 a 15 mm lateral à saída do VII nervo craniano no forame estilomastóideo.	Paralisia facial.
Nervo IX (glossofaríngeo)	Emerge do crânio a partir do forame jugular. Segue medialmente ao processo estiloide e lateralmente ao músculo estilofaríngeo e à artéria carótida interna. Segue inferiormente posterior ao músculo estilofaríngeo. Anteriormente, localiza-se entre os músculos constritores da faringe (superior e médio).	Perda da sensibilidade 1/3 posterior da língua; diminuição da secreção da glândula parótida; síndrome de Vernet; síndrome de Collet-Sicard.
Nervo X (vago)	Emerge do crânio a partir do forame jugular. Cursa posteriormente e medialmente à artéria carótida interna na porção superior. Cursa entre as artérias carótidas comum e jugular interna na porção inferior. Ramo laríngeo superior interno: inferior até a membrana tireo-hióidea, raiz da língua, região epiglótica e túnica mucosa da laringe. Ramo laríngeo superior externo: segue junto ao músculo constritor inferior da faringe até o músculo cricotireóideo.	Paralisia das cordas vocais e disfagia (paralisia dos músculos levantador do palato mole e constritor da faringe); síndrome de Vernet; síndrome de Collet-Sicard.
Nervo XI (acessório)	Emerge do crânio a partir do forame jugular. Cursa entre a inserção do ventre posterior do músculo digástrico no processo mastoide e o músculo esternocleidomastóideo.	Escápula alada e queda do ombro (paralisação dos músculos trapézio e esternocleidomastóideo); síndrome de Vernet; síndrome de Collet-Sicard.
Nervo XII (hipoglosso)	Emerge do crânio a partir do forame hipoglosso, com trajeto ao longo da borda inferior do ventre posterior do músculo digástrico.	Paralisia e atrofia unilateral da língua.
Artéria carótida comum	Direita: ramo do tronco braquiocefálico. Esquerda: ramo do arco da aorta. Segue superiormente e posteriormente à articulação esternoclavicular. Normalmente bifurca na altura da borda superior da cartilagem tireoide (C3/C4).	-
Artéria carótida externa	Segue em sentido ântero-superior posteriormente à mandíbula e profundamente ao ventre posterior do músculo digástrico e estilo-hióideo.	-
Artéria carótida interna	Segue superiormente dentro da bainha carotídea. Anterior aos processos transversos das vértebras cervicais superiores.	-
Veia jugular interna	Localiza-se posterior à artéria carótida interna e aos nervos glossofaríngeo, vago e acessório até entrar na bainha carotídea, Localizada internamente ao músculo esternocleidomastóideo.	-
Veia jugular externa	Formada no interior da glândula parótida. Trajeto descendente profundo ao músculo platisma e superficial ao esternocleidomastóideo.	-

### Avaliação pré-operatória

No caso de traumatismos, as decisões relevantes quanto à abordagem da ACI devem considerar a estabilidade hemodinâmica e lesões concomitantes^32^. Em pacientes que necessitam de intervenções imediatas, o cirurgião pode antever a necessidade de acessos mais amplos com base em alguns critérios, tais como ferimentos por arma de fogo e/ou lesões entre o ângulo da mandíbula e a base do crânio (zona cervical III). Por outro lado, em pacientes estáveis e sem sinais isquêmicos, a avaliação por angiotomografia é relevante e auxilia no planejamento cirúrgico^31^^,^^32^.

### Considerações sobre via aérea e posição do paciente

Após o estabelecimento da via aérea (VA), o paciente é colocado em posição supina, com os braços ao longo do corpo, hiperextensão e rotação contralateral do pescoço, facilitando a dissecção mais distal da ACI^21^^,^^34^^,^^63^. Entretanto, movimentos excessivos podem resultar em compressão das artérias vertebrais ou da carótida contralateral^64^, além de dificultar a retração do ECM por aumentar a tensão do músculo e restringir a mobilidade da ACC e da bifurcação carotídea^65^.

A VA cirúrgica e a intubação nasotraqueal (INT) aumentam o número de técnicas alternativas possíveis para a exposição do SDACI ou da BCA, tornando a subluxação mandibular temporária (SMT) uma opção viável. Na vigência de uma intubação orotraqueal (IOT), a SMT só é possível caso o paciente seja edêntulo ou tenha grandes perdas dentárias. Outra alternativa é realizar a conversão submentual do tubo orotraqueal (TOT) (Figura 2); no entanto, essa manobra acrescenta cerca de 15 minutos ao tempo operatório.

**Figura 2:** Conversão submentual da intubação orotraqueal (IOT). **(A)** Paciente submetido a IOT; **(B)** Acesso submentual; **(C)** Dissecção do soalho bucal; **(D)** Conversão submentual da IOT.

Deve-se ressaltar que a simples manutenção da cavidade oral fechada após a INT não oferece aumento de exposição no acesso à ACI^66^. Entretanto, a associação da INT com a tração superior do complexo maxilomandibular (*chin-up position*), utilizando fitas adesivas ou outros dispositivos, é uma estratégia já descrita para o aumento da exposição da ACI^67^^,^^68^, porém torna-se desnecessária quando se executa a SMT.

### Acessos e manobras utilizados para abordagem da ACI ou BCA

A abordagem do SDACI ou da BCA é alvo de debates na literatura em relação à elaboração da melhor sequência de manobras para promoção progressivamente distal da ACI. As manobras descritas estão resumidas nas Tabelas S1 e S2 (disponíveis como arquivo *on-line* suplementar).

Primeira etapa (acesso convencional): retração do músculo ECM (para lesões até nível de C2)

O primeiro passo para o acesso da ACI/bifurcação carotídea consiste em incisões cutâneas que variam quanto às dimensões e extensões, dependendo do planejamento das etapas subsequentes de exposição. A maioria dos autores descreve uma incisão vertical, iniciando no processo mastoide e seguindo paralelamente ao músculo ECM^1^^,^^3^^,^^9^^-^12^,^^15^^,^^17^^,^^21^^,^^30^^,^^31^^,^^33^^,^^36^^,^^40^^,^^48^^,^^52^^,^^53^, o que permite a exposição do nervo auricular maior, do processo mastoide e da fossa retromandibular^11^. Por outro lado, extensões pré-auriculares também foram descritas^4^^,^^9^^,^^37^, com a vantagem de facilitar a identificação do nervo facial. Quando há o planejamento de osteotomias mandibulares (OMD), as extensões podem ser realizadas ao longo das regiões submandibular, submentual e lábio inferior^12^^,^^14^^,^^30^^,^^34^^,^^37^^,^^39^^,^^43^.

Além dessas estruturas, ramos cutâneos do plexo cervical, como o nervo auricular magno e o nervo cervical transverso, que cursam profundamente até a fáscia do músculo ECM, também podem ser lesados durante esse acesso, resultando em parestesia do lóbulo da orelha e da face anterior do pescoço^5^.

Em seguida, a maior parte dos autores orienta a dissecção ao longo da borda anterior (medial) do ECM para retração lateral^3^^,^^5^^-^17^,^^21^^,^^23^^,^^25^^-^28^,^^32^^-^38^,^^40^^,^^44^^-^50^,^^52^^-^57. A veia facial deve ser ligada, e a alça do hipoglosso pode ser seccionada, expondo toda a porção cervical da ACC e uma média de 26,95 mm (variação de 15 a 45 mm)^5^ do segmento proximal da ACI, equivalente ao nível do terço superior de C2^8^^,^^30^. Por outro lado, dissecções por vias posteriores também foram descritas, como o acesso retrojugular^30^ e pela fossa retromandibular^10^.

Deve-se salientar que é possível aumentar a exposição da ACI com algumas variações da técnica convencional, como a secção/desinserção do ECM ao nível do processo mastoide^7^^,^^8^^,^^18^, a secção seguida de retração^30^^,^^31^^,^^51^ ou a utilização do retrator de Thompson^54^, que permite o controle vascular a 1 cm da base craniana.

Segunda etapa: abordagem do VPD (para exposição adicional que não ultrapassa o bordo superior da C2)

Após a retração/secção/desinserção do ECM, orienta-se a dissecção da face posterior da glândula parótida na região do estilomastóideo, onde a fáscia temporoparietal é seccionada após a identificação e dissecação cuidadosa do VII nervo craniano. Essa dissecção retroparotídea não gera exposição adicional da ACI, mas proporciona campo cirúrgico para a abordagem do VPD, que representa uma importante restrição ao acesso distal da ACI^5^. A partir da exposição dessa estrutura, há uma divergência entre os estudos publicados. Embora alguns autores orientem apenas a retração do VPD, a maioria orienta a secção do VPD (Tabelas S1 e S2, disponíveis como arquivo *on-line* suplementar) (Figura 3), permitindo um ganho médio de 12 a 14 mm na exposição distal da ACI (a partir do acesso convencional oferecido pela retração do músculo ECM)^5^^,^^6^.

**Figura 3:** Abordagem cirúrgica de lesão traumática por arma branca em zona III cervical esquerda. **(A)** Hematoma cervical, plano cutâneo já suturado; **(B)** Acesso por retração do músculo esternocleidomastóideo e divisão do ventre posterior do músculo digástrico, evidenciando a artéria carótida comum (ACC) e o segmento proximal da artéria carótida interna (ACI). O segmento distal da ACI não está exposto (linha tracejada). Sonda de Foley tamponando o sangramento na base do crânio; **(C)** Exposição do segmento distal da ACI (linha pontilhada), coto distal da ACI clampeado.

Convém mencionar que é possível evitar o VPD utilizando o acesso ao triângulo cervical posterior proposto por Sasaki et al.^31^ Esse processo é executado por meio de uma incisão ao longo da borda posterior do músculo ECM, seguida de divulsão longitudinal e retração do músculo para ambos os lados, possibilitando expor a região correspondente ao corpo vertebral da C1.

Durante a realização dessas etapas, diversas estruturas neurovasculares são observadas no campo cirúrgico, incluindo a veia facial comum; o XII nervo craniano, que cursa ao longo da borda inferior do VPD; o XI nervo craniano, que percorre entre a inserção do VPD no processo mastoide e o músculo ECM e cuja lesão resulta em asa escapular e queda do ombro; e a artéria occipital, que normalmente é ligada para aumentar a exposição da ACI e facilitar a mobilização do XII nervo craniano^5^. Um marco anatômico importante do nervo acessório (XI par) é o seu ponto lateral, localizado cerca de 3 a 15 mm abaixo e lateralmente à margem anterior do processo transverso do atlas^7^. Além disso, a dissecção medial da ACI nesse nível pode lesar o nervo laríngeo superior, causando disfagia e disfonia.

Terceira etapa: SMT ou DAE (para lesões até o nível de C2)

Os próximos limitadores anatômicos para o acesso da ACI ou BCA envolvem a região mandibular posterior e o aparato estiloide. A partir desse ponto, não há um consenso na literatura quanto à sequência de procedimentos para aumentar a exposição distal da ACI ou BCA. Convém mencionar que os estudos experimentais foram realizados em cadáveres, avaliando-se unicamente o grau de exposição alcançado com as manobras, desconsiderando fatores relevantes como tipo de VA, tempo para a tomada de decisão, recursos hospitalares disponíveis e *expertise* do cirurgião.

Alguns estudos experimentais orientam a realização da DAE como etapa sequencial^5^^,^^7^^,^^9^^,^^10^, gerando uma exposição adicional de 10 a 15 mm^5^^,^^9^; a SMT seria realizada apenas se necessária, aumentando a exposição em aproximadamente 8 mm^9^. Por outro lado, Mock et al.^6^, também em cadáver, descreveram a realização da SMT antes da DAE, proporcionando aproximadamente 20 mm de exposição adicional da ACI após a divisão do VPD. Caso necessário, a DAE deveria ser realizada em seguida, aumentando a exposição em 3 mm.

Ao analisar criticamente os aspectos inerentes à SMT e à DAE, entendemos que, caso a SMT seja possível, deve ser realizada como alternativa devido a sua rapidez e maior simplicidade técnica, bem como baixa morbidade (Tabelas 2 e 3). Além disso, caso o cirurgião tenha a *expertise* adequada, a SMT não necessita de um cirurgião bucomaxilofacial para sua realização.

**Tabela 2:** Aspectos relacionados à subluxação mandibular temporária.

**Vantagens**	**Desvantagens**	**Complicações**	**Tempo**
Baixa morbidade. Fácil execução. Ampliação do campo cirúrgico (triangular em retangular). Independente de variáveis como sexo, idade e comprimento do pescoço.	Necessidade de intubação nasotraqueal ou conversão submentual da intubação. Distorção anatômica do campo cirúrgico, com aumento teórico do risco de lesão do nervo hipoglosso. Limitação de ampliação do campo cirúrgico em pacientes com eminência articular muito íngreme e fossa articular profunda.	Risco de luxação patológica da mandíbula; disfunção temporomandibular.	10-15 min

**Tabela 3:** Aspectos inerentes à divisão do aparato estiloide.

**Vantagens**	**Desvantagens**	**Complicações**	**Tempo**
Permite exposição da região da artéria carótida interna recoberta pelo aparelho estiloide. A decisão de realização da manobra pode ser feita no intraoperatório. Pode ser realizada junto à técnica de subluxação mandibular temporária para aumento da exposição.	Potencial risco de lesão do IX nervo craniano e dos ramos faríngeos do nervo vago.	Disfagia	-

A SMT, combinada à retratação do ECM e à divisão da VPD, promove acesso a lesões no SDACI (acima de linha de Blaisdell)^6^^,^^23^^,^^25^^-^28 entre 3 e 4 cm da base craniana^23^^,^^28^ e, quando associada à retração dos músculos estiloides, alcança 1 cm proximal à base do crânio^15^. Caso ainda seja necessária exposição adicional da ACI após a SMT, sugerimos a DAE, evitando, assim, exposição aos riscos cirúrgicos inerentes a essa técnica quando o SDACI e a BCA poderiam ser adequadamente expostos pela SMT.

### SMT

É a técnica descrita na maioria dos estudos para procedimentos eletivos em pacientes com BCA ou necessidade de acesso à região distal da ACI para tratamento de doença aterosclerótica, tumores, displasia fibromuscular, pseudoaneurisma, aneurisma e fístula arteriovenosa, embora também haja relatos de sua utilização no trauma agudo (Tabela S2, disponível como arquivo *on-line* suplementar).

A SMT é um procedimento seguro, rápido (10-15 minutos) e relativamente fácil^3^^,^^9^, em que a subluxação anterior do côndilo (10 a 15 mm) na eminência articular resulta em deslocamento anterior de 20 a 30 mm do ramo mandibular, aumentando a exposição distal da ACI pela transformação de um campo operatório triangular em retangular^3^. Com exceção do estudo de Mock et al.^6^, no qual a SMT foi bilateral, os demais estudos (clínicos e em cadáver) utilizaram a técnica unilateral, sem restrições de exposição carotídea.

Para realizar a SMT, há necessidade de INT ou IOT com conversão submentual. Idealmente, a SMT deve ser realizada logo após o estabelecimento da VA (quando sua necessidade for prevista), embora possa ser executada após os acessos cirúrgico iniciais previamente descritos. Nesse ponto, é válido ressaltar que a SMT produz uma leve distorção da anatomia, fazendo com que o VPD e o nervo hipoglosso se desloquem anteriormente e superiormente, além da rotação medial da bifurcação carotídea. Portanto, a dissecção deve ser mais cautelosa para evitar lesão do nervo hipoglosso^9^.

Há variações técnicas para promover a SMT, incluindo amarrias interdentais com fios de aço (1,0 mm) nos dentes caninos e/ou pré-molares (Figuras 4 e 5); instalação de barra de Erich, pinos de Steinmann ou miniparafusos de titânio (2,0 x 10 mm), que são inseridos diretamente na mandíbula e maxila através da mucosa oral nas regiões de pré-molares e caninos contralaterais, associados aos fios de aço (Figura 6); ou, ainda, amarrias circum-mandibulares, em que um instrumento perfurador é inserido através da pele, como a agulha de Reverdin, para passar os fios de aço ao redor do corpo mandibular, adjacente ao periósteo e atravessando a mucosa bucal^3^^,^^17^^,^^21^. No entanto, com o advento dos miniparafusos de titânio, esta última técnica tem menor aplicabilidade devido a sua maior complexidade de execução. Entre as variações técnicas, a menos complexa é a que utiliza amarrias de fios de aço; nesse caso, realiza-se a torção dos fios de aço, resultando em subluxação unilateral da mandíbula (Figuras 4 e 5). Nessa manipulação, deve-se ter cuidado para não provocar o deslocamento iatrogênico do côndilo para a fossa infratemporal. A posição correta de subluxação condilar pode ser facilmente verificada pela inspeção e palpação do aspecto lateral do côndilo imediatamente abaixo da eminência articular^3^.

**Figura 4:** Técnica de subluxação mandibular com fios de aço. **(A)** Posicionamento do fio de aço no espaço interdental mandibular; **(B)** Posicionamento do fio de aço no espaço interdental maxilar; **(C)** Torção dos fios de aço individualmente; **(D)** Torção dos fios de aço cruzados para subluxação mandibular; **(E)** Côndilo mandibular luxado em vista lateral; **(F)** Côndilo mandibular luxado em vista axial.

**Figura 5:** Aplicação da SMT para abordagem de stent contaminado na artéria carótida interna. **(A)** Infecção fistulizando ao nível da cicatriz cirúrgica; **(B)** Subluxação mandibular com amarrias interdentais; **(C)** Acesso por retração do músculo esternocleidomastóideo e divisão do ventre posterior do músculo digástrico; **(D)** Artéria carótida interna reconstruída com enxerto autólogo (artéria femoral).

**Figura 6:** Técnica de subluxação mandibular temporária (SMT) utilizando parafusos de titânio. **(A)** Perfuração óssea com fresa 1,6 mm; **(B)** Instalação dos parafusos; **(C)** SMT com torção dos fios de aço cruzados; **(D)** Aplicação da SMT para abordagem de doença carotídea aterosclerótica, com auxílio de material de moldagem intermaxilar para estabilização adicional.

No que se refere às complicações, dos estudos clínicos publicados sobre SMT (abrangendo 217 pacientes), 12 relataram ausência de disfunção temporomandibular sintomática no pós-operatório^3^^,^^15^^,^^16^^,^^19^^,^^20^^,^^22^^-^28; um não apresentou informações^18^; e apenas dois^17^^,^^21^ descreveram pacientes com sintomas dolorosos na articulação temporomandibular (ATM) no pós-operatório, sendo que todos os casos apresentaram resolução clínica completa em algumas semanas apenas com analgesia. As Tabelas S2 (disponível como arquivo *on-line* suplementar) e 4 apresentam os estudos e os aspectos práticos relacionados à SMT.

Com o intuito de proteger a ATM de luxações indesejadas, Yoshino et al.^25^ descreveram uma técnica de SMT unilateral com auxílio de um *splint* de resina inserido e fixado em posição durante o pré-operatório, guiando o paciente para a posição subluxada. Apesar de a técnica ser menos invasiva, pode ser de difícil aplicação, necessitando de um especialista e planejamento prévio, o que dificulta sua utilização em emergências.

### DAE

Após a secção ou retração do VPD, outra limitação anatômica consiste no aparato estiloide, que é formando pelo processo estiloide, pelos músculos estilo-hióideo, estilofaríngeo e estiloglosso e pelos ligamentos estilo-hióideo e estilomandibular^5^. Essa região pode ser acessada pela fossa infratemporal ou retromandibular^5^^,^^10^. A literatura orienta secção dos músculos e ressecção do processo estiloide (Figura 7) ou estiloidectomia, mantendo as inserções musculares (Tabela S2, disponível como arquivo *on-line* suplementar).

**Figura 7:** Aplicação das técnicas de retração do músculo esternocleidomastóideo (RECM), divisão do ventre posterior do músculo digástrico (DVPD) e divisão do aparato estiloide (DAE) para abordagem de pseudoaneurisma após dissecção de artéria carótida interna por displasia fibromuscular. **(A)** Planejamento da incisão cervical com extensão pré-auricular; **(B)** Acesso cirúrgico RECM + DVDP e exposição do processo estiloide; **(C)** Ressecção do processo estiloide; **(D)** Reconstrução da artéria CI com enxerto venoso.

Os pontos críticos desse acesso incluem o risco de lesões neurológicas, especialmente do IX nervo craniano, que pode ser encontrado medialmente ao processo estiloide e lateralmente ao músculo estilofaríngeo, cursando medialmente para inervar os músculos da faringe^5^^,^^61^^,^^62^. Para minimizar esse risco, Beretta et al.^5^ propuseram uma dissecção cautelosa da ACI mais posterior ao músculo estilofaríngeo. Além disso, a secção do processo estiloide deve ser realizada em direção ao osso temporal para evitar lesões no VII nervo craniano. Os aspectos práticos relacionados à DAE encontram-se na Tabela 3.

Quarta etapa: OMD (para lesões acima da C1)

Outra alternativa para vencer a limitação mecânica causada pelo ângulo e ramo mandibular consiste nas OMD, que podem ser realizadas isoladamente ou junto com a rotação ou remoção temporária de um segmento mandibular, ampliando a exposição cirúrgica. A quantidade de exposição adicional varia de 10 a 26 mm^5^^,^^6^. Por aumentarem o tempo cirúrgico (cerca de 30 minutos)^40^, as OMD normalmente são realizadas em cirurgias eletivas, como nas extensas ressecções de neoplasias, embora haja relatos da sua utilização no trauma^35^^,^^36^.

A literatura descreve diversas técnicas de OMD, sendo as mais utilizadas a osteotomia horizontal ou vertical do ramo, a osteotomia subcondilar, as osteotomias em corpo, parassínfise ou sínfise mandibular (Figura 8) ou uma combinações delas (Tabelas S1 e S2, disponíveis como arquivo *on-line* suplementar). Independentemente da técnica, são necessárias INT/IOT com conversão submentual/VA cirúrgicas.

**Figura 8:** Aplicação da técnica de osteotomia mandibular para exérese de tumor de corpo carotídeo. **(A)** Incisão cervical com extensão mentual; **(B)** Osteotomia realizada na sínfise mandibular; **(C)** Reconstrução carotídea com enxerto venoso; **(D)** Fixação da osteotomia com miniplacas de titânio de 2,0 mm. **(E)** e **(F)** Paciente no segundo mês de pós-operatório.

Antes de realizar as OMD, recomenda-se que miniplacas de titânio sejam pré-moldadas, fixadas e, depois, removidas. A OMD é realizada, e o segmento distal é retraído lateralmente e rodado superiormente e anteriormente. Durante a síntese, os segmentos ósseos são fixados na posição original. Os aspectos práticos relacionados às OMD encontram-se na Tabela 4.

**Tabela 4:** Variações nas osteotomias mandibulares mais utilizadas para acesso ao segmento distal da artéria carótida interna ou à bifurcação carotídea alta.

**Osteotomia**	**Vantagens**	**Desvantagens**	**Complicações**	**Tempo**
Ramo	O acesso pode ser feito na mesma incisão do acesso para exposição carotídea. Exposição e secção são projetadas para evitar lesões nos nervos facial e mandibular.	Maior tempo cirúrgico.	Infecção pós-operatória e má-oclusão.	-
Subcondilar	Preserva os nervos alveolares inferior e lingual. Evita áreas dentadas da mandíbula.	Maior tempo cirúrgico.	Infecção pós-operatória e má-oclusão.	-
Anterolaterais	Acesso mais amplo.	Maior tempo cirúrgico.	Infecção pós-operatória e má-oclusão; cicatriz mais visível no mento e lábio; exodontia prévia do dente relacionada à linha da osteotomia; alto risco de lesão do nervo alveolar inferior.	-

### Proposta de protocolo para abordagem das lesões de ACI ou BCA

Após a revisão das principais manobras descritas para abordagem das lesões do SDACI ou da BCA, demonstradas na Figura 9, constata-se falta de clareza quanto à melhor sequência para exposição em cada caso. Há diversos fatores que auxiliam o cirurgião na seleção ordenada das técnicas, incluindo a *expertise* profissional, especialmente para as técnicas de SMT e OMD, que necessitam de conhecimentos mais específicos, tempo disponível para a tomada de decisão e recursos hospitalares. Esses fatores impactam principalmente a técnica de OMD, que aumenta substancialmente o tempo cirúrgico e requer miniplacas e parafusos de titânio, além da subjetividade do próprio cirurgião ao ponderar as vantagens e desvantagens de cada técnica. Dessa forma, para facilitar a decisão do profissional, propomos o seguinte protocolo (Figura 10).

**Figura 9:** Representação das técnicas de acesso ao segmento distal da artéria carótida interna (ACI); planejamento por imagens de tomografia computadorizada processadas no *software* Mimics® 24.0 (Materialise, Leuven, Belgium). **(A)** Acesso por retração do músculo esternocleidomastóideo (RECM); **(B)** Acesso por RECM e divisão do ventre posterior do músculo digástrico (DVPD); **(C)** Acesso por RECM + DVPD e divisão do aparato estiloide (DAE); **(D)** Acesso por RECM + DVPD + DAE e subluxação mandibular temporária (SMT); **(E)** Acesso RECM + DVPD + DAE e osteotomia mandibular; **(F)** Demonstração do nível de exposição da ACI de acordo com a técnica.

**Figura 10:** Algoritmo proposto para abordagem das lesões em segmento distal da artéria carótida interna (SDACI) e bifurcação carótida alta (BCA). DAE: divisão do aparato estiloide; ECM: esternocleidomastóideo; EI: espaços interdentais; INT: intubação nasotraqueal; IOT: intubação orotraqueal; OMD: osteotomias mandibulares; R/DVPD: retração ou divisão do ventre posterior do músculo digástrico; SM: submentual; SMT: subluxação mandibular temporária; VA: via aérea. * Identificou necessidade de exposição adicional da ACI. ** Quando houver espaço suficiente para acomodar o tubo orotraqueal.

Inicialmente, deve-se determinar o nível da lesão de forma objetiva por meio de angiotomografia em pacientes estáveis ou de forma presumida através de critérios anatômicos em paciente instáveis. A partir de então, sugere-se a seguinte sequência:

1. Lesões altas (cuja necessidade de acesso ampliado foi identificada no pré-operatório): VA recomendada por INT ou IOT + conversão SM ou VA cirúrgica
  1. Lesões ao nível de C2: realizar o acesso ao ECM + retração ou divisão do VPD (R/DVPD) e, se houver necessidade de exposição adicional, proceder com SMT (preferencialmente) ou DAE;
  2. Lesões ao nível da C1: realizar SMT + acesso ao ECM + R/DVPD e, se houver necessidade de exposição adicional, proceder com DAE, seguida por OMD se necessário;
  3. Lesões acima da C1: realizar SMT + acesso ao ECM + R/DVPD + DAE e, se houver necessidade de exposição adicional, realizar OMD. Caso a SMT não seja uma opção, realizar acesso ao ECM + R/DCPD + DAE + OMD;
2. Lesões cuja necessidade de acesso ampliado foi identificada no intraoperatório: analisar a VA
  1. IOT: realizar acesso ao ECM + R/DVPD e, se houver necessidade de exposição adicional, avaliar a possibilidade de conversão SM da IOT ou se há espaços interdentais amplos para a acomodação do TOT. Caso positivo, proceder com SMT, seguida por DAE, combinação dessas manobras e OMD. Caso negativo, a única manobra acessível é a DAE;
  2. INT ou VA cirúrgica: realizar o acesso ao ECM + R/DVPD e, se houver necessidade de exposição adicional, proceder com SMT (preferencialmente) ou DAE, seguida pela combinação dessas manobras e OMD.

Vale ressaltar que, em algumas situações, após a exposição cirúrgica adequada da lesão, pode ser realizado o controle do refluxo da ACI por meio da insuflação de balonetes e cateteres ou *shunts* que, além de permitirem a hemostasia, mantêm o fluxo contínuo na ACI.

## CONCLUSÃO

O acesso cirúrgico à região distal da ACI ou BCA é desafiador, especialmente nos cenários em que a anatomia é distorcida (hematoma, sangramento, aneurisma, tumores), aumentando as dificuldades e potenciais morbidades das manobras necessárias. Entre as manobras descritas, parece razoável que as duas etapas iniciais sejam a abordagem ao músculo EMC, seguida pela secção/retração do VPD. A SMT torna-se um recurso adicional e preferível à DAE pelo seu menor potencial de morbidade. Exposições ainda mais amplas podem ser obtidas com as OMD.

## Material Suplementar

Este artigo acompanha material suplementar.

Tabela S1. Estudos experimentais que descrevem protocolos de acesso cirúrgico ao SDACI ou à BCA.

Tabela S2. Estudos clínicos que descrevem protocolos de acesso cirúrgico ao SDACI ou à BCA.

Este material está disponível como parte da versão online do artigo na página: https://www.scielo.br/j/jvb
